# When increasing vegetable production may worsen food availability gaps: A simulation model in India

**DOI:** 10.1016/j.foodpol.2023.102416

**Published:** 2023-04

**Authors:** Marie L. Spiker, Joel Welling, Daniel Hertenstein, Suvankar Mishra, Krishna Mishra, Kristen M. Hurley, Roni A. Neff, Jess Fanzo, Bruce Y. Lee

**Affiliations:** a Nutritional Sciences Program and Department of Epidemiology, University of Washington School of Public Health, Seattle, WA, United States; b Global Obesity Prevention Center (GOPC), Johns Hopkins University, Baltimore, MD, United States^1^; c Pittsburgh Supercomputing Center, Pittsburgh, PA, United States; d eKutir, India; e Johns Hopkins Bloomberg School of Public Health, Department of International Health, Baltimore, MD, United States; f Johns Hopkins Bloomberg School of Public Health, Department of Environmental Health and Engineering, Baltimore, MD, United States; g Johns Hopkins Bloomberg School of Public Health, Center for a Livable Future, Baltimore, MD, United States; h Johns Hopkins University, Berman Institute of Bioethics, Baltimore, MD, United States; i Johns Hopkins University, School of Advanced International Studies, Washington, DC, United States; j PHICOR (Public Health Informatics, Computational, and Operations Research), City University of New York Graduate School of Public Health & Health Policy (CUNY SPH), New York City, NY, United States; k CATCH (Center for Advanced Technology and Communication in Health), City University of New York Graduate School of Public Health & Health Policy (CUNY SPH), New York City, NY, United States; l AIMINGS (Artificial Intelligence, Modeling, and Informatics for Nutrition Guidance and Systems) Center, City University of New York Graduate School of Public Health & Health Policy (CUNY SPH), New York City, NY, United States

**Keywords:** Food security, Nutrition, Value chains, Vegetables, Discrete event simulation, Odisha

## Abstract

Translating agricultural productivity into food availability depends on food supply chains. Agricultural policy and research efforts promote increased horticultural crop production and yields, but the ability of low-resource food supply chains to handle increased volumes of perishable crops is not well understood. This study developed and used a discrete event simulation model to assess the impact of increased production of potato, onion, tomato, brinjal (eggplant), and cabbage on vegetable supply chains in Odisha, India. Odisha serves as an exemplar of vegetable supply chain challenges in many low-resource settings.

Model results demonstrated that in response to increasing vegetable production 1.25-5x baseline amounts, demand fulfillment at the retail level fluctuated by + 3% to −4% from baseline; in other words, any improvements in vegetable availability for consumers were disproportionately low compared to the magnitude of increased production, and in some cases increased production worsened demand fulfillment. Increasing vegetable production led to disproportionately high rates of postharvest loss: for brinjal, for example, doubling agricultural production led to a 3% increase in demand fulfillment and a 19% increase in supply chain losses. The majority of postharvest losses occurred as vegetables accumulated and expired during wholesale-to-wholesale trade.

In order to avoid inadvertently exacerbating postharvest losses, efforts to address food security through agriculture need to ensure that low-resource supply chains can handle increased productivity. Supply chain improvements should consider the constraints of different types of perishable vegetables, and they may need to go beyond structural improvements to include networks of communication and trade.

## Highlights

• This study conducted discrete event simulation of vegetable supply chains in India.
• The model included spatial, demographic, agricultural, expenditure, and other data.
• Simulated increases in production did not close gaps in vegetable availability.
• In some cases, increased production led to even lower availability due to losses.
• To avoid exacerbating postharvest losses, vegetable supply chains need attention.

## 1 Introduction

### 1.1 Motivation

The supply chains that connect food producers to consumers are critically important for a multitude of goals including food and nutrition security, reduction of postharvest loss and waste, and livelihoods along the value chain. Vegetables are nutritionally important, high-value agricultural crops that face an especially perilous journey through low-resource supply chains due to their perishability. Globally, it has been estimated that 42% of all of fruit and vegetable calories produced are ultimately lost or wasted (Lipinski et al., 2013). In India, as in many in low- and middle-income countries (LMICs), vegetables face supply chain infrastructure challenges including inadequate cold storage, suboptimal packing materials, transportation in open-air trucks on unimproved roads, and open-air wholesale and retail markets (Maestre and Poole, 2018, Maestre et al., 2017, Robinson and Humphrey, 2015, FAO, 2019). Combined with limited information sharing between supply chain intermediaries that may lead to shipping delays in hot and humid climates, these conditions make vegetables particularly susceptible to postharvest loss (Balaji and Arshinder, 2016). Postharvest losses represent missed opportunities for nutrition and agricultural livelihoods (Spiker et al., 2017), as well as a loss of the financial, natural, and other resources invested in improving yields (Kummu et al., 2012).

### 1.2 Objective

The dynamic nature of food supply chains requires methods designed for complexity (Zhao et al., 2019, Veldhuizen et al., 2020, Ridoutt et al., 2019). This work used HERMES Agrifood (the Highly Extensible Resource for Modeling Event-Driven Agricultural Supply Chains), a geospatially explicit discrete event simulation model adapted from HERMES (Pittsburgh Supercomputing Center), which has been used to model supply chains for vaccines and medical products in low resource settings in Benin (Brown et al., 2014, Haidari et al., 2015, Lee et al., 2015), Mozambique (Haidari et al., 2016, Haidari et al., 2017, Lee et al., 2016), Niger (Assi et al., 2011, Assi et al., 2013, Haidari et al., 2013, Haidari et al., 2013, Lee et al., 2012, Lee et al., 2012), Thailand (Lee et al., 2011, Lee et al., 2011), and the state of Bihar in India (Lee et al., 2017, Lee et al., 2019). The research presented here represents a novel adaptation of HERMES to the context of agricultural and food supply chains in Odisha, India.

The objective of this work was to assess how vegetable supply chains in low-resource settings respond to increased vegetable production, using a simulation model that represented vegetable availability and losses under baseline conditions (i.e., reflecting real-world levels vegetable production) and experimental scenarios of increased vegetable production. The model simulated the movement of five key vegetables–potato, onion, tomato, brinjal (eggplant), and cabbage–through village markets, wholesale markets, and urban and rural retailers in the state of Odisha, India.

### 1.3 Policy relevance

This work examines scenarios of increased vegetable production. Vegetable production in India is on an upward trajectory—total vegetable production doubled between 2002 and 2017 (Government of India, Ministry of Agriculture & Farmers Welfare, Horticulture Statistics Division, 2017)—and this trajectory is likely to continue, even if at a slower pace, given remaining yield gaps and the importance of vegetables for livelihoods and food and nutrition security. As an example of yield gaps, average potato yields in Odisha and India overall were 16.7 and 21.2 metric tons per hectare in 2018 (Government of India, Ministry of Agriculture & Farmers’ Welfare, National Horticulture Board, 2015), respectively, while agronomic studies conducted on newly released potato breeds in Odisha suggest potential yields up to 32 metric tons per hectare (Orissa University of Agriculture and Technology (OUAT), Directorate of Research, 2018). Increased production of TOP crops (tomato, onion, and potato) in India has been driven primarily by increased agricultural land use rather than productivity (Tiwari et al., 2021). In addition to closing yield gaps, increases in vegetable production are motivated by concerns about diet quality. India is facing a triple burden of undernutrition, micronutrient deficiencies, and chronic diet-related diseases (Meenakshi, 2016). Poor diet quality—which is characterized by, among other things, inadequate intake of vegetables and fruits—was estimated to account for 17.6% of deaths in India as of 2017 (Afshin et al., 2019). The global vegetable supply is not adequate to meet human nutrition needs (Bahadur et al., 2018, Siegel et al., 2014), and this global situation is reflected in India where the average vegetable supply of 266 g/person/d falls short of the 400 g/person/d recommended by the World Health Organization (Mason-D'Croz et al., 2019). Mason D’Croz notes that while the gap between supply and nutritional needs may widen with population growth and climate change, the per capita availability of fruits and vegetables in South Asia may grow to 615–1,335 g/d by 2050 (Mason-D'Croz et al., 2019), suggesting continued increases in the overall volume of vegetables entering the supply chain. Although there may be factors that disincentivize individual farmers from higher production—for example, the risk of low product prices in response to greater supply (Ali and Kapoor, 2008, George, 2014)—the likely upward trajectory of overall vegetable production reinforces the importance of understanding whether supply chains in low-resource settings can handle increased volumes of perishable foods.

Despite the importance of vegetables as high-value, nutrient-rich crops, policy and research efforts have typically not prioritized horticultural crops (Keatinge et al., 2011, Keatinge et al., 2016, Sanchez, 2020, Haddad, 2020, Kholová et al., 2021). Whereas the CGIAR system—which has traditionally focused on crop improvement for staple grains—has an annual budget of $920 M/year, the World Vegetable Center has an annual budget of $20 M/year for horticultural crop improvement (Chaudhary et al., 2018). Though the CGIAR system has expanded its mission beyond staple crop productivity to include “multiple social and environmental goals,” (Kholová et al., 2021) decades of collective focus on staple crop productivity by a multitude of stakeholders have had lasting impacts (Sanchez, 2020, Haddad, 2020). In addition to a focus on staple grains over horticultural crops, policy efforts have typically focused on crop productivity over postharvest management (Benton and Bailey, 2019). It has been estimated that less than 5% of global agricultural research funding is allocated to postharvest research (Kader, 2003). Despite investments in postharvest infrastructure for horticultural crops from the Government of India, most existing infrastructure is oriented towards staple crops, and most horticultural crop infrastructure is oriented towards high-value, export-quality fruits and vegetables (Thow et al., 2018) which only comprise a small fraction of production (as of 2018, India exports 0.8% of its potato production, 7.7% of its onion production, 0.4% of its tomato production, and 1.2% of other vegetables) (Food and Agriculture Organization, 2021).

In parallel to the agricultural focus on staple grain productivity, nutrition policies have typically focused more on calories than on diet quality. Keatinge and colleagues refer to this as a focus on food security, noting that a focus on nutrition security would prioritize both caloric sufficiency *and* diet quality through horticultural crops, legumes, and animal source foods (Keatinge et al., 2011, Keatinge et al., 2016). India’s National Food Security Act of 2013, also known as the Right to Food Act, takes two main approaches to food security: the Public Distribution System enables low-income households to purchase rice, wheat and millet at subsidized prices at Fair Price Shops, and the Integrated Child Development Services (ICDS) and Mid Day Meals programs take a life course approach that includes direct provisioning of cooked meals for pregnant and lactating women and children through 14 years of age (Tanksale and Jha, 2015). Though some states have started to include pulses and vegetables within the Public Distribution System (Thow et al., 2018), the focus is on cereal grains. Accessing grains through the Public Distribution System is not related to improved child nutritional status (Bartell et al., 2021, Desai and Vanneman, 2015) though some analyses suggest that saving money on staples through the PDS may enable families to purchase more vegetables (Kishore and Chakrabarti, 2015). At Anganwadi Centres where women and children receive ICDS services, nutritional requirements for cooked meals focus on caloric and protein sufficiency (Government of India, Ministry of Women and Child Development, 2009). At schools where children receive Mid Day Meals, nutritional requirements also include vegetables: as of 2006, meals need to contain at least 100 g cereal grains, 20 g pulses, and 50 g of non-tuber vegetables. However, program evaluations show that while the program reaches over 90% of schoolchildren in many states, the pulse and vegetable content of school meals is low and can vary with inflation of food prices (Ramachandran, 2019). It is worth noting that in India, as in my settings, in comparison to starchy staples, vegetable prices are both higher (Headey and Alderman, 2019) and more volatile (Birthal et al., 2019, Sekhar et al., 2018).

Improving India’s vegetable supply is desirable for many reasons; however, crop productivity alone may not necessarily translate into improved food and nutrition security. An important question is whether supply chains with limited infrastructure and coordination can actually handle increased volumes of nutritionally important, perishable foods such as vegetables. Though the United Nations Sustainable Development Goals call for a doubling of agricultural productivity for small-scale farmers (United Nations, 2015), the importance of the value chains that connect producers to consumers–which some have called “the missing middle” (Veldhuizen et al., 2020)–has been overlooked. This research provides policy-relevant insights for this “missing middle” through granular information at the subnational level that integrates a diversity of empirical data through a simulation model that captures the geographically and temporally dynamic nature of food supply chains.

### 1.4 Theory

This work explores the dynamics of supply chains for perishable foods in low-resource settings through a simulation model, specifically a discrete event simulation. The discrete event simulation model used here is dynamic rather than static; it contains stochastic elements with some degree of randomness, rather than being purely deterministic; and it defines system state with a discrete rather than continuous set of values. This type of model is well suited for vegetable supply chains which involve dynamic flows of people, equipment, products, and information that vary geographically and temporally. Ivanov notes that supply chain modeling involves multiple frameworks including a general systems framework that draws from systems science, control theory, and operations research; an integrated modeling framework that combines specific modeling methods; and a computational framework in which algorithms are used to represent supply chain processes through code (Ivanov, 2018).

Simulation models represent the behavior of a system under different conditions with a goal of understanding how system performance changes when certain key parameters are changed; this is fundamentally distinct from the goals of optimization modeling (e.g., linear programming) or statistical modeling (e.g., regression analysis) (Ip et al., 2013, Lund et al., 2017). Because simulation modeling draws from a distinct theoretical approach, its methodological norms differ as well. For example, when evaluating the magnitude of differences between simulation scenarios, frequentist statistical hypothesis testing is typically not performed, because “significant” p-values can be achieved artificially by simply increasing the number of iterations per scenario (White et al., 2014). The value of simulation modeling is not in determining causality or statistical significance, but in exploring scenarios that would not be feasible or cost-effective to test in the real world, such radically changing a supply chain’s structure or the volume or parameters of products flowing through it. In this way, simulation, optimization, and statistical modeling are not in competition with each other, but serve highly complementary functions.

## 2 Methodology

The Highly Extensible Resource for Modeling Event-Driven Agricultural Supply Chains (HERMES Agrifood) is a discrete event simulation model custom built in Python to represent the dynamics of food supply chains. HERMES Agrifood was developed through an iterative process that included planning, data acquisition, model development, validation, and experimental simulation (Pooch and Wall, 1992). The model explored the movement, availability, and loss of five vegetables—potato, onion, tomato, brinjal (eggplant), and cabbage—through agricultural supply chains in the state of Odisha, India. This section provides a high level overview of the model’s mechanisms and parameterization; see **Supplemental Materials** for more detail. This work was classified as exempt by the Johns Hopkins Bloomberg School of Public Health Institutional Review Board (IRB00007503).

### 2.1 Study setting

This model represented vegetable supply chains in the state of Odisha, India. Odisha faces supply chain challenges common across South Asia: the agricultural marketing system is largely informal, with limited infrastructure for storage and transportation of horticultural crops (Mittal, 2007, Sharma, 2012, Negi and Anand, 2015, Rais and Sheoran, 2015). Cold storage infrastructure is especially scarce and can only accommodate a small fraction of horticultural crops; it is estimated that an additional 3.27 million metric tons of cold storage would be needed to close this gap (National Centre for Cold Chain Development, 2015). Odisha has a population of 44 million, with 83% living in rural areas and 62% working in the agricultural sector (India Census Bureau, Office of the Registrar General & Census Commissioner, Ministry of Home Affairs, Government of India, 2011). With an average farm size of 1.04 ha, 83% of the state’s producers are smallholder farmers (Government of Odisha, Directorate of Agriculture & Food Production, 2014). Odisha has ten agro-ecological zones; its climate is warm and humid, and agricultural production depends on monsoon rains as the state lacks perennial rivers. Odisha’s primary agricultural products are rice (46% of land under cultivation) and pulses (23%), with vegetables (including potatoes) comprising 8% of land under cultivation (Government of Odisha, Directorate of Agriculture & Food Production, 2014). Based on a comparison of production and consumer expenditure data, Odisha procures the majority of its potatoes from other states and produces a surplus of onion, tomato, brinjal, and cabbage (Government of India, Ministry of Agriculture & Farmers’ Welfare, National Horticulture Board, 2012, Government of India, Ministry of Agriculture & Farmers’ Welfare, National Horticulture Board, 2014). Geographic and seasonal variation in the production of these crops in Odisha are shown in section 6.4 of the Supplemental Materials; while some crops (such as tomato and brinjal) are harvested during almost all months, potato and onion are only harvested during 2–3 months of the year. Growth in Odisha’s agricultural sector has been driven by land use expansion (Keatinge et al., 2016) (with 41% of land under agricultural cultivation), as crop yields in Odisha remain inefficient compared to India overall (Pattanaik and Mohanty, 2016). As in most of India, where the organized retail sector accounts for only 5% of food sales nationwide (Panneerselvan, 2012), consumers in Odisha purchase vegetables primarily through the informal retail sector.

### 2.2 Model description

This research used HERMES Agrifood, a geospatially explicit discrete event simulation model custom built in Python based on the HERMES model for vaccine supply chains (Pittsburgh Supercomputing Center). Discrete event simulation is commonly used to describe the dynamics of queues, and a food supply chain can be thought of as a series of queues. Each discrete event—for example, the movement of food or trucks from one location to another—represents a change in the state of the system. Based on model mechanisms and parameter values that reflect real-world supply chains, simulation runs capture discrete events and generate summary outcomes describing supply chain performance (Haidari et al., 2017). Model outcomes explored here include *demand fulfilment*, which describes the proportion of instances in which vegetables were available at supply chain locations when simulated consumers arrived; *total loss*, which describes vegetable loss as a proportion of the total amount that entered the supply chain; and *time through the supply chain*. Each of these outcomes can be disaggregated by supply chain level, geographic location, and vegetable.

### 2.3 Supply chain structure

#### 2.3.1 Supply chain levels

**Fig. 1:**
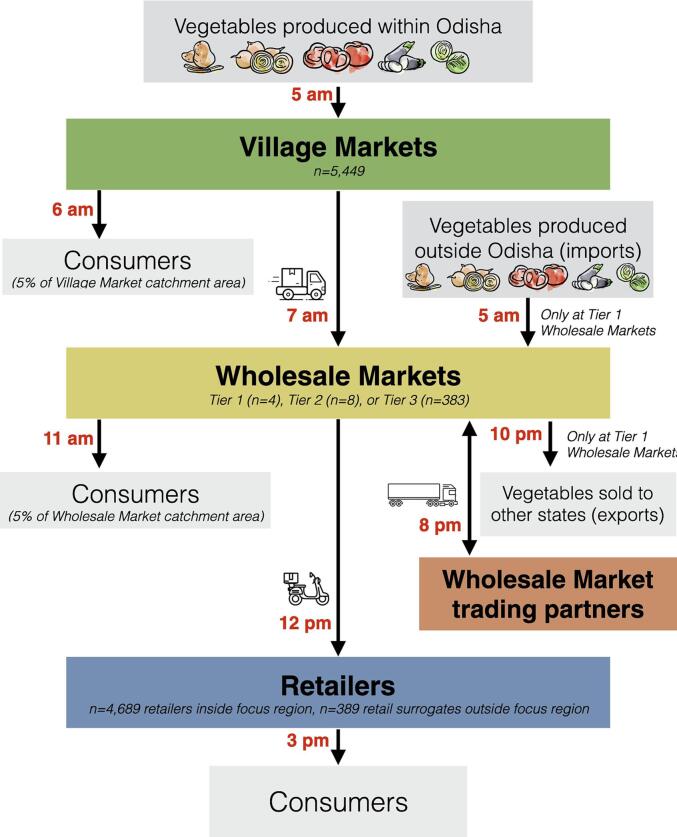
**Supply Chain Structure.** Vegetables entered the supply chain at village markets or wholesale markets, and they exited the supply chain when consumers purchased them (at village markets, wholesale markets, or retailers) or when they underwent loss before they could be purchased (through either expiration of breakage, where breakage refers to losses from other factors such as mishandling or accidental spills). The times shown (e.g., 5am, 3 pm) represent average times, with actual vehicle departures or consumer arrivals drawing from a Poisson distribution around these average times.

As shown in Fig. 1, the model included three distinct supply chain levels: village markets, wholesale markets, and retailers. Intermediaries between these levels were represented implicitly through transportation routes. Farm-level production was represented implicitly by amounts of vegetables entering the supply chain. The model’s 395 wholesale markets represented *mandis*, or agricultural markets run by the Agricultural Produce Market Committee (APMC). While APMC markets are not the sole market channel for farmers—alternatives include contract growing; farmer producer companies or cooperatives; and direct sales to traders, retailers, or consumers outside of mandis—most vegetables produced in Odisha pass through wholesale markets, often mediated by local traders represented here by the supply chain level of village markets (Sharma, 2012). It is estimated that 60% of produce in India is marketed through traditional wholesale market value chains (Gulati et al., 2022).

#### 2.3.2 How vegetables entered the supply chain

Simulated vegetables entered the supply chain through village markets in amounts that reflected estimates of horticultural crop production from the Government of India’s National Horticulture Board (Government of India, Ministry of Agriculture Farmers’ Welfare, National Horticulture Board, 2012), geographic patterns that reflected block-level agricultural production data from the Government of India’s Agriculture Census (Government of India, Agriculture Census Division, 2011), and temporal patterns that reflected monthly variation between peak, lean, and off-seasons (Government of India, Ministry of Agriculture & Farmers’ Welfare, National Horticulture Board, 2014). All crop production was assumed to be marketed surplus, rather than production for own-consumption. Amounts of vegetables entering the supply chain were reported on the basis of mass, which was converted to volume using crop-specific bulk densities (Sharan and Rawale, 2003) as HERMES Agrifood packs storage devices by volume.

#### 2.3.3 How vegetables exited the supply chain

Vegetables exited the supply chain if they were purchased by consumers at village markets, wholesale markets, or retailers; or if they were lost due to breakage or expiration before they could be purchased. *Consumer purchase* of vegetables occurred according to population catchment areas for each location (Center for International Earth Science Information Network - CIESIN - Columbia University, 2016) and per capita demand for each crop (Government of India, Ministry of Statistics and Programme Implementation, National Sample Survey Office, 2012). *Breakage* refers to product loss from typical conditions of transportation, packaging, and handling (e.g., bruising, accidental spills). The breakage rates were Poisson-distributed with the mean breakage rates set at 2% at each supply chain level or transport leg. *Expiration* refers to product losses that occurred when a product reached the end of its lifespan before being purchased by a consumer or broken. Because crops had longer lifespans in cold storage, time spent in cold storage slowed products’ aging rates. Expiration therefore represented both suboptimal temperature storage conditions and an excessive amount of time in the supply chain. Vegetable losses—through either breakage or expiration—could occur during storage or transport.

#### 2.3.4 Trade between supply chain levels

Supply chain locations were connected by transportation routes, and each route was assigned a vehicle with a specific capacity and was characterized by an ordering policy. Ordering policies were defined by the quantity of product shipped (fixed or variable), the frequency of shipment (fixed or variable), whether the supplier delivered the product or the recipient would fetch the product, and whether the shipment was executed a single time or persistently until the order was fulfilled.

**Fig. 2:**
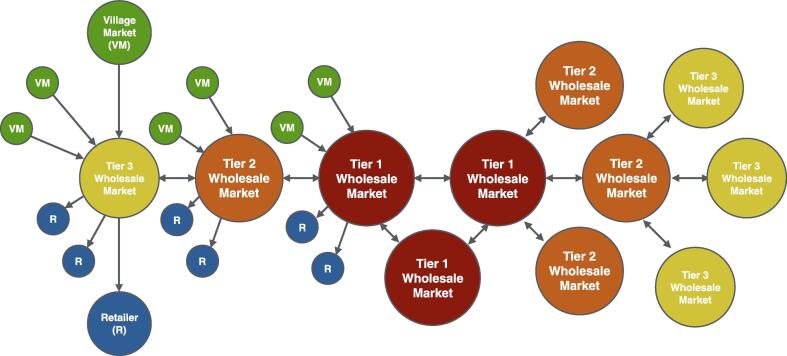
**A three-level supply chain structure where bidirectional trade between wholesale markets linked geographic areas.** The left half of the figure illustrates how a single wholesale market could simultaneously be supplied by many village markets while also supplying many retailers. The right half of the figure illustrates the tiered structured through which wholesale markets engaged in bidirectional lateral trade.

A vegetable’s path through the three-level supply chain was determined by both geographic proximity and consumer demand. Vegetables from village markets were transported to the closest wholesale market, and retailers retrieved vegetables from the closest wholesale market. The supply chain had bidirectional trade because the 405 wholesale markets traded with each other. Not every wholesale market traded with every other wholesale market; the markets traded in the hierarchical structure shown in Fig. 2 in which wholesale markets were classified as Tier 1 (n = 4), Tier 2 (n = 8) or Tier 3 (n = 395) markets. Whereas Tier 3 markets had a single wholesale trading partner, Tier 1 and Tier 2 markets were high-volume markets that aggregated products regionally and traded with each other based on consumer demand to bridge geographic areas. During simulation runs, information was shared between markets on a daily basis, with estimates of downstream demand communicated to upstream markets to trigger shipments and determine their size.

### 2.4 Model boundaries

The model included five vegetables: potato, onion, tomato, brinjal (eggplant), and cabbage. These vegetables were chosen because they are nutrient-rich, non-staple crops; they are commonly produced in Odisha^a^; they are commonly traded in Odisha, comprising an average of 39% of transactions at wholesale markets^b^; and they represent a range of supply chain constraints affecting horticultural crops (e.g., potato can be transported in sacks stored for months, whereas tomato requires more delicate packaging and has a shorter lifespan). These crops are a focus of India’s National Horticulture Mission, and TOP vegetables (tomato, onion, and potato) are the three largest produced and consumed vegetables in India, as well as crops for which India is the world’s second largest producer (Tiwari et al., 2021, Gulati et al., 2022).

The geographic boundaries of the model were the state boundaries of Odisha. Odisha has approximately 44 million residents and contains 30 administrative districts, 315 administrative blocks, and over 50,000 villages (Government of India, Ministry of Home Affairs, India Census Bureau, 2011). To reflect trade across state boundaries, the model used state-level imports and exports to create a state of approximate equilibrium, in accordance with the seasonality of vegetable production: crops not produced sufficiently within Odisha during any given month were “imported” from other states in amounts that would help meet typical consumer expenditures, and crops produced in excess of typical consumer expenditures were “exported” to other states. In other words, on any given simulated day, the supply chain as a whole contained sufficient quantities of vegetables to meet Odisha’s average consumer demand. The model included individual representations of all known village markets (n = 5,449) and wholesale markets (n = 407) across Odisha. Because representing all individual retailers (upwards of 110,000) would have made simulation run times infeasible, across most of the state products leaving any given wholesale market proceeded to a single surrogate location representing the total retail-level demand of that market’s catchment area, rather than proceeding to individual retailers. To allow for a more detailed exploration of supply chain dynamics across all three supply chain levels, individual retail locations (n = 4,689) were represented within the “focus region,” a 35-kilometer radius around the city of Bhadrak, representing a catchment area of 2.7 million people (see Fig. S1).

### 2.5 Data sources and model validation

**Fig. 3:**
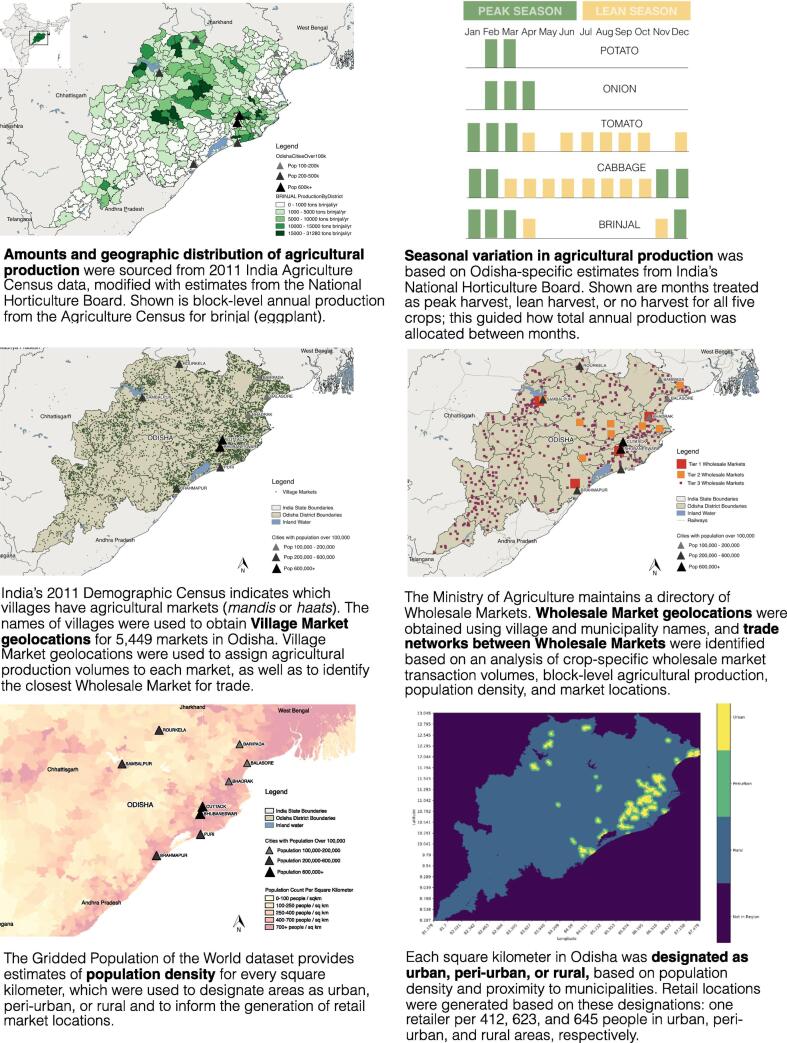
Visualization of a subset of model inputs. Where maps are shown, they depict the state, district, and block boundaries of Odisha, India. See Methods section and Supplemental Information for more detail on how these model inputs were sourced and utilized.

HERMES Agrifood represents operational aspects of the supply chain using real-world data to populate the model’s parameter values. Extant data sources, detailed in Table S1, included peer-reviewed literature, government publications and databases (including national and state level data), technical reports, and spatial and demographic data. For each model parameter, a literature search was conducted and sources were selected on the basis of the authoritativeness of the source, rigor of data collection methods, recentness, and relevance to the context of vegetable supply chains in Odisha. Data sources were triangulated against each other, and in some cases, multiple data sources were integrated to inform a model mechanism or a specific set of parameter values. As shown in Table S1, data sources ranged from 2009 to 2017. Data inputs were compiled between 2015 and 2019 and simulation runs were conducted 2018–2019. Extant data sources were supplemented by field observations in Odisha and input from expert stakeholders in order to contextualize and assess the quality of data on supply chain processes. Observations were conducted at 13 sites including farms, rural markets for vegetable aggregation, wholesale markets, and retail locations in the Khurda and Nayagarh districts of Odisha in 2016. During observations, a translator was present to facilitate conversation with supply chain stakeholders including farmers, traders, drivers, and retailers. Data were recorded through field notes and memos written immediately following observations, and members of the research team periodically conducted peer debriefing. Fig. 3 visualizes a subset of model inputs and the **Supplemental Materials** provide more detail on data sources, parameter values, and model assumptions.

HERMES Agrifood differs from the original HERMES model not only in the nature of the data that populate the model, but also in the expansion of certain model mechanisms to reflect the characteristics of agricultural supply chains. One key addition to HERMES Agrifood is the ability to support bidirectional trade within a supply chain level—in this case, between wholesale markets—which entails calculating the demand at each location so that markets with lower inventory can trigger shipments from trading partners with higher inventory. HERMES Agrifood includes an expanded mechanism to represent product waste in which vegetables degrade at a linear rate until they reach their maximum lifespan. The model also supports the ability to prioritize more perishable items in storage and transportation (e.g., if there is limited space on a truck, less perishable items may be left behind) and to prioritize the sale of products to one type of population over another (e.g., local consumers can be prioritized over trade to other wholesale markets). HERMES Agrifood also enables seasonal variation in both vegetable production and vegetable demand.

Model verification and validation took place throughout all project phases. During planning, data acquisition, and model development, face validity was established in multiple phases by sharing and seeking feedback on narrative, numerical, and visual representations of the model’s mechanisms and assumptions with the project team and expert stakeholders. Experimental simulation was conducted in multiple phases—first with more geographically limited versions of the model, and then with versions that included inputs for all of Odisha—and simulation outputs were shared at each phase to assess face validity of the results. Model verification, or the process of establishing that the intended conceptual description of the model is reflected in the model’s implementation, was conducted repeatedly throughout model development and experimental simulation process. As one example, to verify that the timing of transportation routes or wholesale trade requests reflected model inputs and intended mechanisms, detailed stock curves showing the hourly inventory at a set of specific supply chain locations were examined after multiple model runs, each with different inputs. To test the model’s criterion validity, certain data sources not used to parameterize the model were used as a comparator against output from simulation runs. For example, in simulation runs, the volume and timing of vegetables passing through wholesale markets was based on block-level production data from the agricultural census, geographic proximity of markets, ordering policies, consumer demand, and other model mechanisms. The simulated amount of vegetables passing through wholesale markets was compared to empirical data on wholesale market transaction volumes, which were *not* used to parameterize the model; Fig. S7 shows that empirical and simulated market volumes were highly correlated, suggesting that the parameter values and simulated trade network reflected real world mechanisms.

### 2.6 Experimental scenarios

Model runs were conducted to simulate the system at baseline, as well as five experimental scenarios in which the amount of vegetables entering the supply chain was multiplied by factors of 1.25, 1.5, 2, 3, and 5. Simulated increases in vegetable production with multiplying factors of 1.25 or 1.5 represent more realistic increases in vegetable production that could happen over a time frame of 5, 10, or 15 years, given that India’s total vegetable production doubled between 2002 and 2017 (Lee et al., 2017). Although a 2-, 3-, or 5-fold increase in vegetable production is unlikely within a relatively short period of time, simulating increases of this magnitude enabled exploration of supply chain dynamics under more extreme variation.

Simulated increases in production were applied assuming no other changes in the supply chain. Though it is possible that over a period of 5, 10 or 15 years in which agricultural production increases there are accompanying improvements in supply chain infrastructure, it is also possible for supply chain improvements to be stagnant for years at a time. For example, although the estimated total capacity of cold storage in India increased six-fold between 2004 and 2014, of approximately 7,000 cold storage facilities constructed during this time, approximately 1,200 were closed or non-functional as of 2014 due to issues with maintenance and electricity (National Centre for Cold Chain Development, 2015). Additionally, although Odisha comprises 1.83% of India’s total population, as of 2014 it had only 1.0% of the country’s total cold storage capacity.

The results for each experimental scenario represent the average simulation results from multiple iterations. Each iteration had a “burn-in period” of seven simulated days (i.e., the time it takes to transition from an “empty” supply chain to a supply chain in regular operation) and a subsequent time horizon of one simulated year.

## 3 Results

### 3.1 Characteristics of the system under baseline conditions

The modeled representation of Odisha’s supply chain included 5,449 village markets (with direct sales to 5% of the local population, averaging 390 local consumers each (SD = 377)) and 405 wholesale markets (with direct sales to 5% of the local population, averaging 5,464 local consumers each (SD = 5,202)). Village markets and wholesale markets served primarily to aggregate products further along in the supply chain, with the remaining 90% of vegetable purchases taking place at the retail level. Each retailer served an average of 412, 623, or 645 people in urban, peri-urban, and rural areas respectively. The supply chain as a whole served a total simulated population of 44 million residents per day.

As vegetables moved through the supply chain, the average travel time was 27 min between village markets and wholesale markets, 5 h between wholesale markets, and 24 min between wholesale markets and retailers. The average time through the supply chain (i.e., the time between entering the supply chain at the village market or wholesale market and exiting the supply chain due to consumption or loss) was 1.5–1.9 days depending on the vegetable, with maximum times of 25.3 days for potato, 20.3 days for onion, 11.3 days for tomato, 11.3 days for cabbage, and 7.8 days for brinjal. Vegetables spent the greatest proportion of time at wholesale markets, given that products could accumulate at wholesale markets or be traded between wholesale markets multiple times.

Under baseline conditions, daily wholesale market arrivals for all vegetables (sum of potato, onion, tomato, brinjal, and cabbage) ranged from 9,499 to 10,041 tons at Tier 1 markets (average of 9,692 tons); 1,909 to 8,193 tons at Tier 2 markets (average of 3,294 tons); and 1 to 395 tons at Tier 3 markets (average of 52 tons). As an example of vegetable composition at wholesale markets of different sizes, at Tier 1 markets, 42% of arrivals (by tons) were potatoes, 15% were onion, 13% were tomatoes, 17% were brinjal, and 13% were cabbage. At Tier 3 markets, 16% of arrivals (by tons) were potatoes, 11% were onions, 22% were tomatoes, 33% were brinjal, and 18% were cabbage. These averages span all time points within the simulated year, and they reflect that brinjal was abundantly produced throughout Odisha and traded among the smaller Tier 3 wholesale markets whereas potato was dependent on trade from other states, first passing through the larger Tier 1 or Tier 2 wholesale markets before reaching retailers.

### 3.2 Under baseline conditions, the supply chain did not fulfill demand

The outcome of *demand fulfillment* describes the proportion of instances in which vegetables were available at a supply chain location (i.e., village market, wholesale market, or retailer) when a simulated consumer arrived. Demand fulfillment is specific to each crop and can be reported for individual locations or as an average across categories. Under baseline conditions, the Odisha-wide average demand fulfillment at the retail level was 67% for potato, 69% for onion, 82% for tomato, 89% for brinjal, and 72% for cabbage. In other words, cabbage was available during 72% of instances when simulated consumers arrived at retailers, averaging across all retail locations and simulated days.

### 3.3 Under baseline conditions, 22–36% of vegetable production was lost

The outcome *total loss* describes vegetable loss as a proportion of the total amount that entered the supply chain, on the basis of physical volume. Total loss included two mechanisms: *expiration* (when a vegetable’s time in the supply chain exceeded its lifespan) and *breakage* (loss from other factors such as mishandling and accidental spills). For potato, for example, 25% of all potato production was lost, including 17.5% of total production lost due to expiration and 7.5% of total production lost due to breakage. Most of the remaining 75% was sold to consumers (inside or outside of Odisha), with negligible amounts remaining in stock in the supply chain at the end of the simulation. Other losses included 22% of all onion production (including 16% of the total from expiration), 25% of all tomato (including 21% of the total from expiration), 36% of brinjal (including 32% of the total from expiration), and 23% of cabbage (including 19% of the total from expiration). Because breakage rates across all vegetables were less variable—they drew from a Poisson distribution with an average of 2% loss per supply chain stage or transport leg, totaling 4–8% losses of total production—losses due to expiration were more informative about supply chain performance as they reflected additional time in the supply chain.

Disaggregating total losses by supply chain stage, an average of 0.3–1.9% of total production was lost in storage at village markets depending on the vegetable, 0.3–0.8% was lost in transport from village markets to wholesale markets, 12.3–24.4% was lost in storage at wholesale markets, 4.9–10.5% was lost in transport between wholesale markets, 0.01–0.05% was lost in transport between wholesale markets and retailers, and 0.03–0.11% was lost in storage at retailers. Overall, the majority of vegetable losses occurred in connection with wholesale markets—either in storage at wholesale markets, or in transportation between wholesale markets. These losses were distributed across wholesale markets of all sizes and were not disproportionately driven by the largest wholesalers (for example, the three Tier 1 wholesalers accounted for 35% of total wholesale arrivals yet only 20% of total wholesale losses). Across all supply chain stages, a greater proportion of total loss occurred during storage (13–28% of total production depending on the vegetable) than during transportation (7–11% of total production). Storage losses were higher for more perishable vegetables (28%, 21%, and 16% of total tomato, brinjal, and cabbage production, respectively) than for less perishable vegetables (14% and 13% for potato and onion, respectively).

### 3.4 Increasing production did not close the gap in demand fulfillment

Simulated increases in vegetable production by factors of 1.25x and 1.5x represented more realistic increases in vegetable production that could happen over a time frame of 5, 10, or 15 years, whereas simulated increases by factors of 2x, 3x, and 5x were used to explore the dynamics of the supply chain under more extreme variation.

**Fig. 4:**
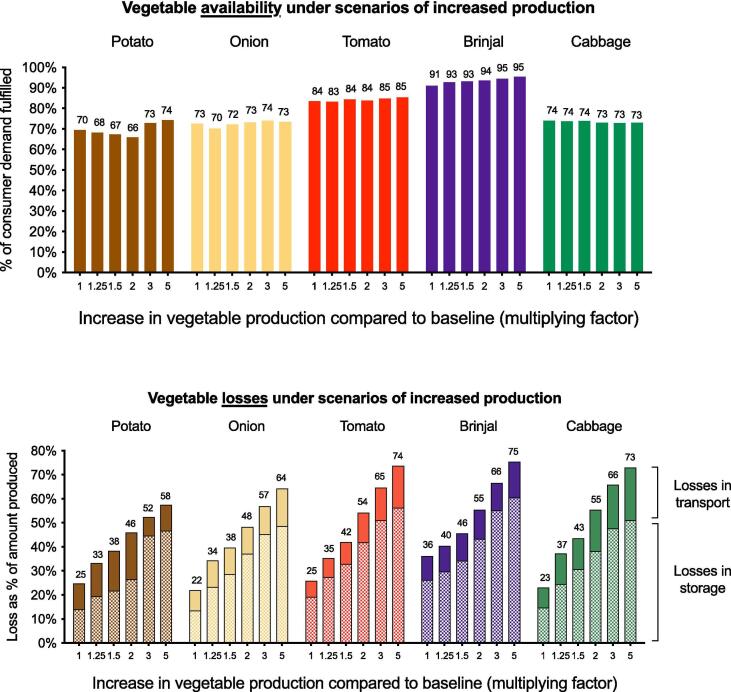
**Vegetable availability and losses under scenarios of increased production.** The top panel shows availability as a percentage of consumer demand fulfilled at the retail level. The bottom panel shows total postharvest loss as a percentage of total production, and total losses are further disaggregated by the proportion of loss that occurred during storage or transport.

Increasing the amount of vegetables entering the supply chain either led to modest improvements, no improvements, or decrements in retail-level demand fulfillment ranging from + 3% to −4% demand fulfillment (Fig. 4). Even under the most extreme increases in production (5x), demand fulfillment only increased modestly, if at all; demand fulfillment for brinjal, for example, only increased 4% over baseline conditions (from 91% to 95%), whereas demand fulfillment for cabbage decreased 1% from baseline conditions (from 74% to 73%).

### 3.5 Increasing production led to disproportionately high rates of postharvest loss

Increasing the amount of vegetables entering the supply chain led to consistent and substantial increases in total loss. Even under a modest 1.25x increase in production, there was an additional 4–14% increase in total loss compared to baseline conditions, depending on the vegetable. Under a 2x increase in production, there was an additional 16–31% increase in total loss, and under a 5x increase in production there was an additional 32–49% increase in total loss compared to baseline conditions.

Fig. 4 shows total losses as well as the proportion of loss during storage or transport, demonstrating that the majority of loss typically occurred during storage. Furthermore, as production increased, storage losses tended to increase while transport losses were more consistent, indicating that more extreme increases in production led to greater storage losses. Combined with the fact that the majority of losses occurred from the mechanism of expiration rather than breakage, these storage losses indicate that vegetables tended to accumulate and expire in the supply chain under conditions of increased production.

**Fig. 5:**
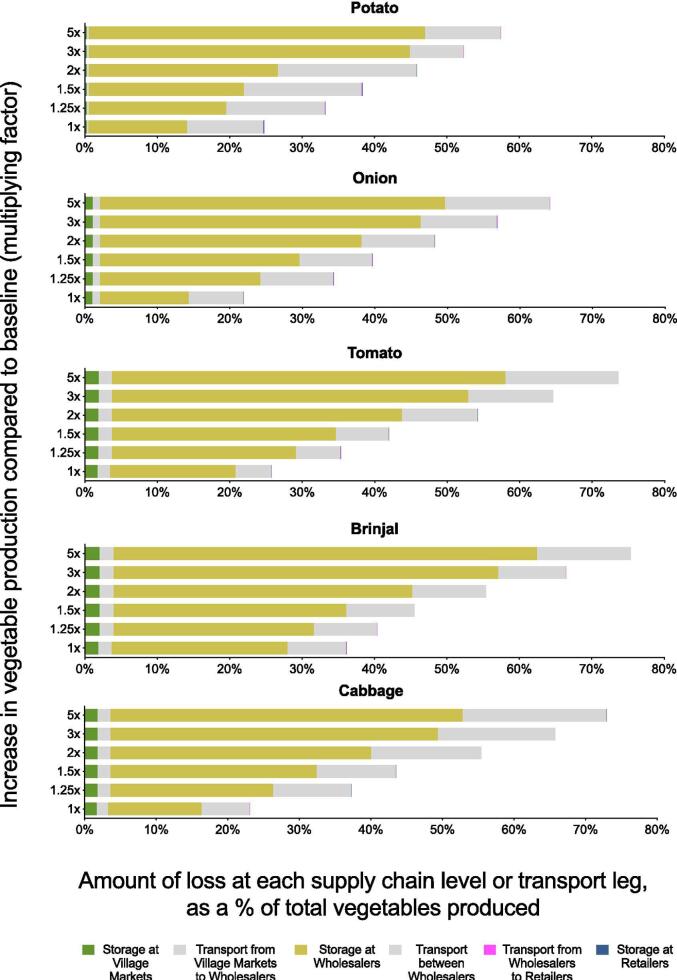
**Composition of vegetable loss throughout the supply chain.** In this figure the composition of total vegetable loss is broken down by stage of the supply chain, including both storage and transport between supply chain levels. The proportions represent the amount of loss at each stage divided by the total amount produced in the supply chain as a whole; for example, of the total amount of potato that entered the supply chain, 25% of total production was lost throughout the supply chain as a whole, and 14% of total production was lost specifically in storage at the Wholesale Market level, while negligible amounts were lost after the wholesale stage.

Fig. 5 disaggregates total loss by supply chain stage. Across all scenarios, the majority of loss occurred in storage at wholesale markets or in transport between wholesale markets. For example, under conditions of 2x production, 36% of total production was lost at wholesale markets (compared to 13% at baseline) and 15% was lost in transit between wholesale markets (compared to 7% at baseline), whereas trivial amounts were lost at village markets or in transport out of village markets (<2% at 2x and baseline) and at retailers or in transport to retailers (<1% at 2x and baseline). Because wholesale markets traded with each other, products could pass through multiple wholesale markets before being purchased directly by consumers, transported to retailers, or expiring.

## 4 Discussion and policy implications

Findings from a geospatially explicit discrete event simulation model demonstrated that vegetable supply chains in Odisha were unable to meet consumer demand due to high rates of postharvest loss. These losses were driven by accumulation and expiration at the wholesale market level, they were exacerbated under conditions of increased production, they differed depending on the perishability of vegetables, and they were driven by a modeled system structure that reflected real-world gaps in information-sharing and emergent trade. This work informs policies that seek to improve vegetable availability and decrease vegetable losses in low-resource settings. Specifically, efforts to increase horticultural production without improving supply chain infrastructure and coordination may exacerbate supply chain losses; postharvest management efforts should take into account the specific constraints of different types of perishable vegetables; and infrastructure investments may be more fruitful if complemented by improved networks of communication and trade between supply chain players.

### 4.1 Policy Implication #1: Policies that seek to improve vegetable availability need to consider supply chains, not just crop production. Without supply chain improvements, efforts to improve horticultural yields may inadvertently exacerbate postharvest losses

Simulation results showed that supply chain dynamics caused gaps in vegetable availability under baseline conditions, even for vegetables with sufficient baseline production. Despite the food supply containing sufficient amounts to meet average consumer demand, baseline demand fulfillment ranged from 70 to 91%, depending on the vegetable. Why, for example, would the baseline demand fulfillment for brinjal only be 91% even though baseline brinjal production was equivalent to 5.2x Odisha’s typical household expenditures on brinjal? As a thought exercise, a supply chain with sufficient agricultural production and a very simple structure–say, a single village market supplying a single nearby retailer–should result in 100% demand fulfillment, barring any losses from transport, handling, vehicle failures, or other unexpected events. As the supply chain becomes more complex–with multiple levels, communication between levels relying on individual supply chain intermediaries, thousands of locations with varying infrastructure, long travel times, and bidirectional trade–there are more opportunities for vegetables to accumulate, exceed their lifespans, and fail to reach the specific retail locations where they are sought by consumers.

Simulation results also demonstrated that postharvest losses were exacerbated under conditions of increased production, especially for more perishable vegetables. One might expect that increasing the amount of vegetables entering a supply chain would translate into a proportional improvement in availability; for example, doubling agricultural production might double the amount of product available for consumers. What is notable about rates of demand fulfillment across simulated scenarios is not just that they fell short of *proportional* increases–for example, even in the case of a five-fold increase in brinjal production, demand fulfillment only increased 4% over baseline conditions–but that in some scenarios increasing vegetable production actually resulted in *lower* demand fulfillment, owing to additional accumulation and expiration at intermediate supply chain stages. One can visualize supply chain losses as water leaving a leaky hose. The simulated increases in vegetable production were akin to sending a larger volume of water through the hose; the increased rates of postharvest loss and decrements in availability seen with higher vegetable production might be conceptualized as pressure in the hose causing additional leakage. In this model, even the smallest simulated increase in production—a 25% increase—resulted in disproportionate increases in postharvest losses. This indicates that in the absence of other changes to the system, vegetable supply chains in this context had limited capacity to absorb increased production.

Policy efforts aiming to improve vegetable availability need to consider supply chains in order to avoid exacerbating postharvest losses. Inadequate vegetable availability, as well as downstream indicators such as inadequate vegetable purchase or consumption, may not represent inadequate crop production. Gaps in availability may also stem from postharvest losses related to supply chain constraints, especially in low resource settings where the majority of food losses are at the production and supply chain levels. In this study, across all vegetables and scenarios of increased production, whereas demand fulfillment only varied by 3–4% in either direction, increases in postharvest loss were quite substantial: doubling production led to 38–43% of total production lost through expiration or breakage, representing additional losses of 10–20% over baseline conditions.

The finding that increased horticultural crop production could exacerbate postharvest losses without even improving consumer availability is notable because crop productivity is actively promoted as a goal of agricultural policy and investments in rural development (The World Bank, 2012, Shroff and Kajale, 2008). The focus on productivity is logical given projections that yields will need to double by the year 2050 (Ray et al., 2013, Tilman et al., 2011). In light of these estimates, many research agendas center high yield technologies, development of new crops and cropping systems, delivery and utilization of crop inputs, and other strategies to close yield gaps (Tilman et al., 2011, Cassman and Grassini, 2020). The FAO estimated that achieving these productivity increases in LMICs would require an average annual net investment of USD 83 billion (Food and Agriculture Organization, 2009). They note this estimate includes both “primary agriculture and downstream support services.” The analysis presented here reinforces the need for these downstream support services, especially those that can reduce supply chain losses of horticultural crops. Without accompanying investments in infrastructure and coordination for postharvest management, interventions to help farmers close yield gaps may inadvertently exacerbate already high rates of postharvest losses.

### 4.2 Policy Implication #2: Strategies to reduce vegetable losses may benefit from addressing the constraints of different types of perishable vegetables

Across all simulated scenarios of production, higher rates of loss for tomato, brinjal, and cabbage (which lasted 4–7 days at ambient temperature) compared to potato and onion (which lasted 15–21 days at ambient temperature) reflected their perishability. For example, at baseline, up to 25% of the less perishable vegetables were lost, whereas up to 36% of the more perishable vegetables were lost. Under the scenario of 50% increased production, up to 38% of the less perishable vegetables were lost, whereas up to 46% of the more perishable vegetables were lost. Though brinjal tended to have higher demand fulfillment because it is produced abundantly (brinjal production was 5.2x Odisha’s annual brinjal expenditures), it also had the shortest lifespan at ambient temperature (4 days) and had the highest rates of loss of all vegetables in all experimental conditions.

It is worth noting that while the results here are presented in terms of “more perishable” or “less perishable” vegetables, *all* vegetables are highly perishable in comparison to other foods like grains. Unlike grains which can be stored for years, even longer-lasting vegetables like potato and onion need to be traded or consumed within months or weeks. The perishability of vegetables also poses complications related to seasonality: this model assumed that demand for each vegetable was constant throughout the year, and meeting this demand during off-season months required trade with other states, which meant that vegetables spent more time in wholesale-to-wholesale trade rather than being consumed closer to where they were produced. Potato and onion, for example, though they be stored for longer, are also more seasonal (see Fig. S18) and thus susceptible to storage and transport losses during wholesale-to-wholesale trade despite their longer lifespans.

The finding that perishability drives losses indicates that in food supply chains with limited resources and coordination, it may be important to focus on averting losses of more perishable crops such as vegetables—and especially the most perishable among them—which are typically lost at higher rates. Policies that support postharvest loss reductions such as subsidies or incentives to build or maintain cold storage infrastructure typically do not focus on more perishable crops. The modeled supply chain did not include cold storage capacity, reflecting the current inadequacy of cold storage for vegetables in this setting (National Centre for Cold Chain Development, 2015). Approximately three-quarters of India’s cold storage capacity for non-dairy food items is single-commodity storage used for potatoes exclusively; 23% is multi-purpose storage, of which the share for horticultural crops is unknown; and less than 1% is dedicated to fruits and vegetables (Rais and Sheoran, 2015, Sivaraman, 2016). Odisha has fewer than 100 operational large-scale cold storage facilities for horticultural crops National Centre for Cold Chain Development, 2015, and these facilities are typically used to keep buffer stock of potato and onion for economic reasons rather than to avert postharvest losses (Sivaraman, 2016). India’s 1955 Essential Commodities Act enabled state governments to control the price, storage, and movement of politically sensitive food commodities, including potato and onion, until the Act was amended in 2020. Though potato and onion (along with other foods including grains, pulses, and oilseeds) are no longer subject to these government controls, their prior inclusion in the Essential Commodities Act was due in part to their likelihood of being hoarded, indicating that cold storage facilities are not always used exclusively for postharvest loss reduction (Sivaraman, 2016).

Postharvest loss reduction policies that do not address the varying perishability and needs of different horticultural crops may hinder efforts to avert losses of high-value, nutrient dense crops that are already lost at higher rates. Whereas grain warehouses are tightly controlled by government entities such as the Food Corporation of India (FCI) and the Central and State Warehousing Corporations (CWC and SWC), cold storage of perishable crops is coordinated by a large, diffuse network of smaller private sector players (Sivaraman, 2016). Some policies intended to catalyze the growth of cold storage infrastructure—such as the Government of India’s Foreign Direct Investment (FDI) policy which requires investments to be larger than USD 100 million—target large facilities that are not compatible with the small scale of most cold storage facilities for perishable vegetables. If policy efforts to support the growth of cold storage infrastructure are also intended to reduce losses, they will need to target more perishable crops and the stakeholders who store and transport more perishable crops.

### 4.3 Policy Implication #3: “Supply chain improvements” may need to go beyond structural improvements to include networks of communication and trade

In simulation runs, the majority of postharvest losses occurred in storage at wholesale markets or in transportation between wholesale markets. For example, under baseline conditions, 24% of total potato production was lost at or between wholesale markets and never reached the retail level, and this grew to 45% of total production under conditions of doubled production. These findings reflect Food and Agriculture Organization data on total calories lost and wasted in South and Southeast Asia showing that the supply chain stage with the greatest losses is handling and storage (37%), followed by production (32%) and distribution and markets (15%) (Lipinski et al., 2013). These findings also suggest that postharvest loss in this system was driven by vegetable accumulation and expiration at the wholesale market level, rather than being driven by a lack of storage or transport space (in which we would see vegetables overwhelming the volumetric capacity of storage devices or vehicles) or a supply that exceeds consumer demand (in which we would see high rates of loss at retailers).

The modeled system structure—which reflects real world conditions in which there is a limited network of wholesale communication and trade—contributed to vegetable accumulation and loss at the wholesale market level. Simulated wholesale markets received daily information about the inventory and demand of their trading partners, but—as in the real world—they were not omniscient about the inventory and demand of all wholesale markets in the system beyond their trading partners, nor did they create new trading relationships in order to redistribute surplus vegetables to geographic areas with unmet demand. As a result, although the supply chain was theoretically capable of connecting any combination of geographic locations at the wholesale level—unlimited trade between the 405 wholesale markets would yield upwards of 163,000 trading routes—a finite network of trade without emergent trade relationships resulted in vegetable losses at the wholesale level alongside unmet needs at the retail level. Thus the fact that demand fulfillment did not reach 100% for any crop, even when production was increased up to five-fold, can be attributed in part to a limited network of communication and trade and a lack of emergent trade relationships to redistribute surplus vegetables to areas with unmet demand.

These findings indicate that reducing postharvest losses for perishable foods in resource-limited settings may not be as simple as investing in additional infrastructure such as cold storage or refrigerated transport. Though there is demonstrated need for additional cold storage capacity in India’s horticultural supply chains (Negi and Anand, 2015, National Centre for Cold Chain Development, 2015, Maheshwar and Chanakwa, 2006), reducing postharvest losses may also require improved ways of sharing information and coordinating action throughout the supply chain. India’s Ministry of Agriculture has estimated that 25–30% of horticultural crop losses in India may be due to a lack of postharvest technologies and integration of postharvest storage, transportation, and marketing facilities (Government of India, Ministry of Agriculture, 1999). A study of cold chains for horticultural crops in India found that while the largest inhibitor of efficient cold chains was a lack of adequate infrastructure, supply chain structure and interrelationships between supply chain actors were also important inhibitors (Joshi et al., 2009). Bustos and Moors noted that postharvest losses can be reduced by supply chain collaborations that allow supply chain actors to align incentives, exchange information, and leverage new technologies (Bustos and Moors, 2018). There has been momentum in investments for information and communication technology (ICT) for agriculture in low-resource settings. In addition to focusing on agricultural production (for example, for farm financing or sharing data to improve crop productivity), new ICT developments might focus on issues specific to the supply chain; for example, connecting producers to markets through microentrepreneurs (Dubé et al., 2020), or enabling traders at all scales to act on real-time data on wholesale market inventory or consumer demand (Parwez, 2014, Gokarn and Choudhary, 2021). Investments in low-resource supply chains for perishable vegetables should include investments that strengthen social capital, not just physical infrastructure. In addition to investing in developing new technologies, the usability of these tools among stakeholders and their potential to effectively reduce losses also needs to be evaluated. While this study focused on the most common vegetable supply chain channels in this setting—those involving public wholesale markets—efforts to reduce postharvest losses should consider all types of market channels for perishable vegetables including direct purchase, cooperatives, contract farming, and international exports. Supply chain strategies should also consider solutions from all sectors and scales, including public policies and private sector initiatives at local, state, and national levels.

### 4.4 Limitations, strengths, and generalizability

#### 4.4.1 Limitations

This discrete event simulation modeled supply chain operations; other factors that may affect or be affected by supply chain operations–such as behavioral, economic, and political factors–were beyond the scope of this work. For example, the model did not examine food prices, which can both respond to food supply *and* influence decisions about where or when vegetables are traded or discarded. The scenarios tested here assumed that vegetable demand was consistent with the most recently available consumer expenditure data, rather than changing in response to supply; future work could incorporate Say’s law, which asserts that higher production can create its own demand (Kates, 2005). The scenarios tested here also examine vegetable availability and losses on a physical basis relative to their baseline values; units of value not explored here include servings of food, proportion of nutritional recommendations, or the economic value of crops (taking into consideration current food prices, which vary between the included crops).

Simulated increases in vegetable production were applied assuming no other changes in the supply chain, whereas real-world supply chain stakeholders may respond to increased production with expanded storage capacity, more frequent or higher capacity transportation, emergent trade relationships with other markets, or increased processing or export of surplus products. The fact that the model included a finite set of trading relationships and did not initiate new and emergent connections reflects real world conditions in which supply chain actors are not omniscient about the inventory and demand of all other players in the system and they trade with a limited number of partners. Though it would be possible to design simulations that compare alternative scenarios of information sharing or the creation of emergent networks in response to demand, there is value in understanding the capacity of a current system to absorb increases without further adaptation. Structural improvements, policies, and behavior change can be slow to take hold; Reardon and Minten have noted agricultural marketing system reforms in India have been slow and complicated to implement (Reardon and Minten, 2040).

This model may have under- or over-estimated vegetable losses in some cases, as additional factors beyond the scope of this model can affect losses including subjective perceptions of vegetable quality and the specific timing within a market day. For example, vegetables might be discarded at the end of a market day even if they have not yet reached the end of their lifespan (by not capturing this, this model would under-estimate losses); or, retailers might drop food prices in evening hours to encourage purchases and minimize waste (by not capturing this, this model would over-estimate losses).

As a simulation model of supply chains, HERMES Agrifood examined factors that affect food availability at various points of purchase (including retail, wholesale, and village markets) and the stability of the food supply, but it did not directly examine other aspects of food and nutrition security such as food access, utilization, agency, or sustainability (FAO et al., 2022). This work focused on what happens to vegetables from the time they left the farm as marketed surplus to the time they were made available for consumer purchase, and thus did not examine the impact of crop productivity on the amount or quality of foods production for own-consumption—for example, through kitchen gardens or homestead food production interventions, which are being studied for their potential to contribute to nutritional outcomes (Bird et al., 2019). In the context of home food production for own consumption, supply chain constraints do not interfere with the ability of crop productivity to translate into improved availability; but in the context of purchasing vegetables produced by others, supply chains matter.

#### 4.4.2 Strengths

This work represents the novel adaptation of a discrete event simulation for supply chains to the context of low-resource agricultural supply chains. By representing supply chains with multiple levels and geographic and temporal specificity, this work enables the exploration of detailed supply chain dynamics. This work was strengthened by the use of extensive literature review and stakeholder input to inform the development of model mechanisms, as well as the triangulation of multiple data sources to inform model inputs. Characteristics of the system at baseline reflected real-world vegetable supply chains in Odisha, demonstrating model fidelity. For example, the relative transaction volume at modeled wholesale markets was highly correlated with the distribution of products among real-world wholesale markets in Odisha. Additionally, postharvest losses at baseline ranged from 22% of total onion production to 36% of total brinjal, reflecting estimates of vegetable losses in India overall. Estimates of horticultural crop loss and waste in India have been reported as 4.6–12.5% (depending on the vegetable), from a 2014 national study limited to production losses (Jha et al., 2015); 9–32%, from 2012 field surveys in northeastern states that included production, wholesale, and retail losses (Small Farmers’ Agribusiness Consortium, Ministry of Agriculture and Farmers Welfare, Government of India, 2015, Committee on Doubling Farmers’ Income, Ministry of Agriculture and Farmers Welfare, Government of India, 2017); and 26–27%, from an analysis of 2019 Food and Agriculture Organization data including all supply chain levels (Nuthalapati et al., 2022). Though this mechanistic model was not designed to serve as a postharvest loss prediction tool, the fact that modeled losses reflect trends in empirical estimates lends validity to the model’s mechanisms and parameter values.

Using simulation modeling to understand the complex dynamics of vegetable supply chains provides unique insights that complement other methods such as optimization or frequentist statistical modeling. A simulation model enables the exploration of complex supply chain dynamics through a diverse array of metrics that may not be available at scale through empirical data. For example, while there may be empirical data on the marketplace availability of foods, those data may be infrequently collected or from limited geographic samples. HERMES Agrifood provides a framework to integrate data from various systems including agriculture, demographics, supply chain operations, and dietary intake. A simulation model also enables testing scenarios for which there may be a dearth of empirical observations; for example, news outlets report instances of agricultural gluts overwhelming supply chains, but these gluts are not captured by systematic data collection schemes such as the horticultural census. In the absence of comprehensive empirical data on food supply chains, simulation modeling can leverage existing empirical data from multiple systems to explore complex relationships that affect food security, poverty reduction, and malnutrition in LMICs.

Simulation modeling also enables the exploration of more extreme scenarios such as a doubling or tripling of vegetable production which, while unlikely to occur in the short-term, reveals patterns in how system structure leads to system outcomes. For example, one could imagine a situation in which modest increases in vegetable production do not translate into increased vegetable availability, but more extreme increases do. In this study, however, the more extreme scenarios revealed that even unrealistically high levels of vegetable production were not enough to overcome the constraints of the current system structure, which included a network of finite trade relationships at the wholesale level. The inclusion of these scenarios also revealed non-linear relationships; demand fulfillment for potato, for example, was lower than baseline at 2x production but higher than baseline at 3x production, suggesting that while extreme production increases could compensate for supply chain bottlenecks, more modest increases overwhelmed the system.

#### 4.4.3 Generalizability

In this work, the data used to parameterize the HERMES Agrifood model were informed by empirical data and stakeholder input from Odisha, and so the resulting simulation results are specific to Odisha and can inform subnational policies for this state with 44 million residents. Comprehensive data on model mechanisms, assumptions, and parameters have been provided here and in the **Supplemental Materials**, both to contextualize these particular simulations runs and to serve as a blueprint for how simulation modeling efforts for other settings might integrate a similar resolution and variety of food supply chain data sources. The characteristics of vegetable supply chains in Odisha—including large volumes of perishable vegetables traded without cold chain infrastructure through a diffuse network of traders with multiple intermediaries including large wholesale markets—are shared by some vegetable supply chains throughout India and South Asia, and thus study findings may be generalizable to supply chains with similar characteristics. Exploring the dynamics of vegetable supply chains with markedly distinct structures—for example, settings where certain crops do not pass through public wholesale markets—would require distinct model mechanisms and data inputs. Though findings from any set of simulation runs necessarily reflect the context for which they were designed, the HERMES Agrifood simulation model can be adapted for any product, geography, or supply chain structure for which relevant data and information on supply chains mechanisms are available.

## 5 Conclusions

Findings from a geospatially explicit discrete event simulation model of vegetable supply chains in Odisha, India, suggest that the dynamics of low-resource supply chains can create gaps in vegetable availability for consumers even when vegetable production is theoretically sufficient to meet demand. Simulated increases in agricultural production did not close gaps in vegetable availability because limited communication and trade between supply chain intermediaries resulted in accumulation and loss in some parts of the system alongside unmet demand in others. In this model, more modest increases in vegetable production did not result in meaningful improvements and demand fulfillment, but did lead to disproportionate increases in postharvest losses, indicating that existing vegetable supply chains have a limited capacity to absorb increased production. In some cases, more extreme increases in production actually worsened demand fulfillment because they resulted in substantially higher rates of postharvest loss, which were more pronounced for more perishable vegetables.

These results emphasize the importance of ensuring that supply chains in low-resource settings are prepared to handle increased horticultural crop production. Agricultural research and investments tend to focus on crop yields, but yields alone may not translate into improved food availability. Increases in horticultural crop production are inevitable, and should be welcomed: horticultural crops support rural livelihoods and are important for food and nutrition security, and closing yield gaps can help India prepare for population growth and growing per-capita demand for non-staple foods. The question is whether supply chains with limited infrastructure and coordination are ready to handle these increases when they occur; if they are not, efforts to improve horticultural yields may inadvertently exacerbate postharvest losses. Supply chain improvements should consider the constraints of different types of perishable vegetables, and they may need to go beyond structural improvements to include networks of communication and trade.

Odisha’s vegetable supply chains–which are characterized by inadequate infrastructure and limited information sharing between multiple intermediaries–face challenges common to many resource-limited supply chains throughout South Asia and other settings. In resource-limited settings with high rates of postharvest loss, improving supply chain performance may help to ensure that increased vegetable production actually translates into increased vegetable availability for populations aiming to improve food and nutrition security.

### CRediT authorship contribution statement

**Marie L. Spiker:** Conceptualization, Formal analysis, Investigation, Writing – original draft, Writing – review & editing, Visualization. **Joel Welling:** Conceptualization, Methodology, Software, Validation, Resources, Data curation, Writing – review & editing. **Daniel Hertenstein:** Conceptualization, Methodology, Software, Validation, Resources, Data curation, Writing – review & editing. **Suvankar Mishra:** Conceptualization, Validation, Writing – review & editing. **Krishna Mishra:** Conceptualization, Validation, Writing – review & editing. **Kristen M. Hurley:** Conceptualization, Writing – review & editing, Supervision. **Roni A. Neff:** Conceptualization, Writing – review & editing, Supervision. **Jess Fanzo:** Conceptualization, Writing – review & editing, Supervision. **Bruce Y. Lee:** Conceptualization, Methodology, Resources, Writing – review & editing, Supervision, Project administration, Funding acquisition.

## Declaration of Competing Interest

The authors declare that they have no known competing financial interests or personal relationships that could have appeared to influence the work reported in this paper.

## Footnotes

a The state’s top ten horticultural products, ranked by tons of production, are brinjal (eggplant), tomato, cabbage, cauliflower, okra, pumpkin, onion, sweet potato, potato, and bottlegourd.

b Based on the AgMarknet reporting system for 106 wholesale markets in Odisha in 2017: 39% equals the total annual tons of arrivals for potato, onion, tomato, brinjal, and cabbage by the total annual tons of all commodities (including grains, vegetables, fruits, animal source foods, and fibers).
