# Supplementary material for: When increasing vegetable production may worsen food availability gaps: A simulation model in India
Source: Food Policy. 2023 Apr;116:102416. doi: 10.1016/j.foodpol.2023.102416 (PMC10206406; doi:10.1016/j.foodpol.2023.102416)
Supplement: Supplementary data 1 [file mmc1.docx]

**Supplemental Materials for**

When Increasing Vegetable Production May Worsen Food Availability Gaps: A simulation model in India

**This PDF file includes:**

Supplementary text

Figures S1 to S19

Tables S1 to S10

Supplementary references

Table of Contents

[Supplemental Section 1. Overview of Supplemental Materials and Methods 4](#_Toc117874322)

[Supplemental Section 2. Model boundaries 4](#_Toc117874323)

[2.1 Vegetables 4](#_Toc117874324)

[2.2 Supply chain levels 4](#_Toc117874325)

[2.3 Geographic boundaries 4](#_Toc117874326)

[Supplemental Section 3. Supply chain structure 6](#_Toc117874327)

[3.1 How vegetables entered the supply chain 6](#_Toc117874328)

[3.2 How vegetables exited the supply chain 6](#_Toc117874329)

[3.3 How vegetables were transported through the supply chain 6](#_Toc117874330)

[Supplemental Section 4. Model Runs 7](#_Toc117874331)

[4.1 Time horizon 7](#_Toc117874332)

[4.2 Number of iterations per run 7](#_Toc117874333)

[4.3 Compute resources 7](#_Toc117874334)

[Supplemental Section 5. Model Outcomes 8](#_Toc117874335)

[5.1 Outcomes related to product availability 8](#_Toc117874336)

[5.2 Outcomes related to product loss 8](#_Toc117874337)

[5.3 Outcomes related to supply chain performance 8](#_Toc117874338)

[Supplemental Section 6. Model Inputs (data sources, parameter values, and assumptions) 9](#_Toc117874339)

[6.1 Locations and Storage Devices 12](#_Toc117874340)

[6.2 Routes and Vehicles 23](#_Toc117874341)

[6.3 Vegetable Characteristics 26](#_Toc117874342)

[6.4 Consumer Demand for Vegetables 31](#_Toc117874343)

[Supplemental References 34](#_Toc117874344)

List of Figures

[Figure S1 Boundaries of the state of Odisha and the focus region. 5](#_Toc117875867)

[Figure S2 Locations of village markets in the model. 12](#_Toc117875868)

[Figure S3 Locations of wholesale markets int he model. 13](#_Toc117875869)

[Figure S4 Comparison of wholesale market vegetable arrivals to nearby population size and vegetable production 14](#_Toc117875870)

[Figure S5 Amount of vegetables (potato, onion, tomato, brinjal, and cabbage), traded at wholesale markets within the AgMarknet system. 15](#_Toc117875871)

[Figure S6 Modeled and real-world vegetable arrivals and wholesale markets: Sum of 5 vegetables, shown on a log scale. 16](#_Toc117875872)

[Figure S7 Modeled and real-world vegetable arrivals at wholesale markets. 17](#_Toc117875873)

[Figure S8 Population counts per square kilometer in Odisha. 18](#_Toc117875874)

[Figure S9 Map of area classified as urban, peri-urban, and rural in the model. 19](#_Toc117875875)

[Figure S10 Map of retailers in the focus region. 20](#_Toc117875876)

[Figure S11 Distribution of distances from retailers to the closest wholesale market in urban, peri-urban, and retail areas. 21](#_Toc117875877)

[Figure S12 Wholesale market arrivals of potato, onion, tomato, brinjal, and cabbage in the context of total arrivals (all food commodities) in Odisha in 2017, per AgMarknet data. 22](#_Toc117875878)

[Figure S13 Distribution of travel times by route type. 24](#_Toc117875879)

[Figure S 14 Block-level production according to Agriculture Census data. 26](#_Toc117875880)

[Figure S15 Comparison of per capita production and consumption of vegetables in Odisha. 27](#_Toc117875881)

[Figure S16 Distribution of number of village markets per block 27](#_Toc117875882)

[Figure S17 Seasonal calendar of vegetable harvest in Odisha from the National Horticulture Board. 28](#_Toc117875883)

[Figure S18 weekly wholesale market arrivals in Odisha, season seasons of peak and lean harvest. 29](#_Toc117875884)

[Figure S19 Distribution of population served by types of supply chain locations. 33](#_Toc117875885)

List of Tables

[Table S1. Overview of main data sources used to develop model inputs 9](#_Toc117875886)

[Table S2. Types of wholesale markets in the hierarchical network of lateral trade 15](#_Toc117875887)

[Table S3. Characteristics of areas classified as urban, peri-urban, and rural 19](#_Toc117875888)

[Table S4. Characteristics of retailers in focus region 21](#_Toc117875889)

[Table S5. Description of ordering policies 23](#_Toc117875890)

[Table S6. Vehicle capacities 25](#_Toc117875891)

[Table S7. Vegetable characteristics: demand, bulk density, and lifespan 30](#_Toc117875892)

# Supplemental Section 1. Overview of Supplemental Materials and Methods

This research used the Highly Extensible Resource for Modeling Event-Driven Agricultural Supply Chains (HERMES Agrifood) to simulate the supply chain of a subset of vegetables in Odisha, India. HERMES Agrifood is a geospatially explicit discrete event simulation model (DES) custom built in Python. The model consists of virtual representations of locations, transportation routes, vehicles, storage devices, personnel, products that flow through the supply chain (in this case, vegetables), and demand for products.

This document describes model boundaries, the supply chain structure, how model runs were conducted, outcomes used to assess supply chain performance, and model inputs (including data sources, parameter values, and assumptions related to model attributes).

# Supplemental Section 2. Model boundaries

## 2.1 Vegetables

The model included five vegetables: potato, onion, tomato, brinjal (eggplant), and cabbage. These vegetables were chosen because they are common in the typical cuisine in Odisha; they comprise an average of 39% of transactions at wholesale markets in Odisha^[[1]](#footnote-1)^; and they represent a variety of supply chain constraints related to lifespan, cold storage requirements, and requirements for packaging and handling.

## 2.2 Supply chain levels

The model represented three distinct supply chain levels: village markets, wholesale markets, and retailers. Intermediaries between these levels (e.g., traders and other middlemen) were represented implicitly through transport routes. Farms were not represented explicitly as a supply chain level, though their production was represented implicitly by the amounts of vegetables entering the supply chain through village markets. Each village market, wholesale market and retailer was represented by a single location, though in reality each of these markets may consist of multiple buyers and sellers.

## 2.3 Geographic boundaries

The geographic boundaries of the model were the state boundaries of Odisha. Odisha has a population of approximately 44 million people and contains 30 administrative districts, 315 blocks, and over 50,000 villages.^1^ The model represented village markets (n=5,449) and wholesale markets (n=407) throughout the state of Odisha, and individual retail locations (n=4,689) were represented only within the “focus region” shown in **Figure S1**.

The “focus region” was a 35-kilometer radius (approximately 2 hours of driving time) around the city of Bhadrak, and its retailers served a population of approximately 2.7 million people in urban, peri-urban, and rural areas. Within the focus region, the model included locations for individual retailers. Outside the focus region, populations that would be served by retailers were represented through a single retail surrogate at each wholesale market. The inclusion of Odisha-wide data for the first two supply chain levels allowed for a more accurate representation of Odisha’s statewide horticultural crop production and trading relationships, while the use of a focus region for the last level allowed for an exploration of individual retailers and more feasible simulation run times.^[[2]](#footnote-2)^

Geographic boundaries can create “edge effects” in which the behavior of the system differs for locations close to the geographic edges of the modeled space. For example, vegetables at a village market close to the border of Odisha and West Bengal might be sold to a wholesale market in West Bengal in the real world, but in this simulation they would always proceed to a wholesale market within Odisha. Despite the fact that state-level and other administrative boundaries are “porous” and many transactions occur across these boundaries,^2^ setting geographic boundaries was necessary for the feasibility of model development. Using Odisha’s state boundaries in combination with a focus region at the retail level allowed for alignment with high-quality data sources at the state, district, and block levels.


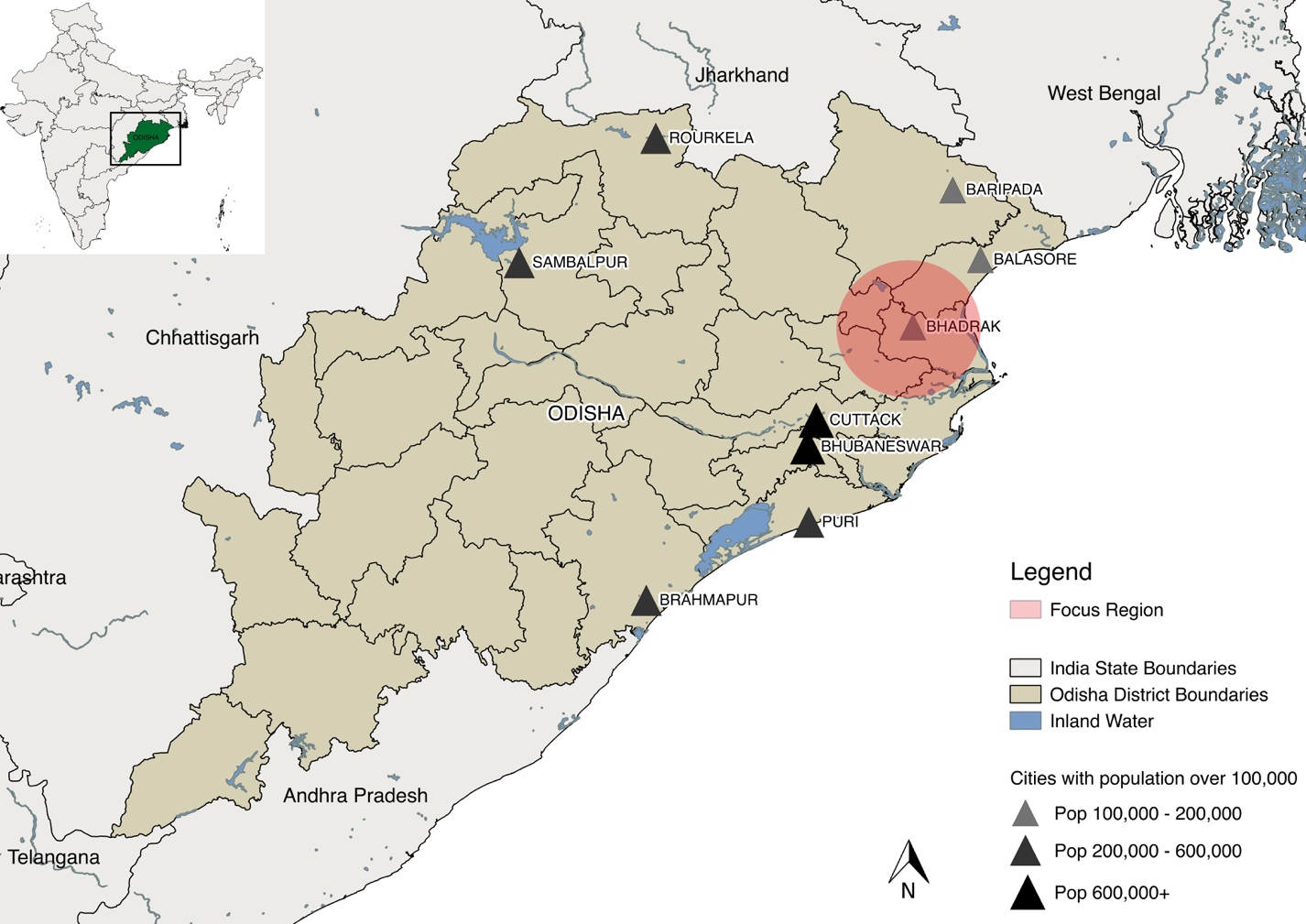


Figure S1 Boundaries of the state of Odisha and the focus region.

For village markets and wholesale markets, individual locations were included throughout the state of Odisha. For retailers, individual locations were included only within the “focus region” shown in the red circle. *Outside of the focus region, populations that would be served by retailers were represented through a single retail surrogate at each wholesale market (see section 6.5.3 for more detail).*

# Supplemental Section 3. Supply chain structure

The model represented three supply chain levels: village markets (n=5,449), wholesale markets (n=405), and retailers (n=4,689). This three-level supply chain structure represents a marketing channel that is common for horticultural crops in Odisha and similar settings based on observations, key stakeholder input, and published characterizations of horticultural crop supply chains within Odisha^3-6^ and in similar settings within India.^7-13^ Common variations on this supply chain structure include additional intermediaries between the producer and wholesaler; additional intermediaries between the wholesaler and retailer; and the presence of cooperatives, processing facilities, or export markets.

## 3.1 How vegetables entered the supply chain

Vegetables produced in Odisha entered the supply chain through village markets in amounts that reflected real-world geographic and temporal trends in horticultural crop production (see Section 6.4 for more detail).

Vegetables produced outside of Odisha (i.e., “imported” from other states in India) entered the supply chain through select wholesale markets in amounts that met average monthly consumer demand (using consumer expenditures as a proxy; see Section 6.5 for more detail). Potato, onion, cabbage, and tomato were imported to help meet consumer demand during 10, 9, 6, and 2 months of the year, respectively. Brinjal was produced in excess of typical consumer demand each month, and thus was never imported.

## 3.2 How vegetables exited the supply chain

Vegetables exited the supply chain if they were purchased by Odisha consumers at village markets, wholesale markets, or retailers; if they were traded to “export” markets outside of Odisha at select wholesale markets; or if they were lost due to breakage or expiration before they could be purchased or exported.

Simulated consumers were able to purchase vegetables at each supply chain level. At each supply chain location, consumer demand reflected population density in relation to each location, the simulated frequency of consumer visits, and typical amounts purchased.

Regarding state-level “exports” to other states, potato was not produced in sufficient quantities to meet Odisha’s demand during any month and thus was never exported. All other crops were exported during at least one month of the year.

Loss of vegetables could occur either during storage or transport, and loss occurred by two mechanisms: breakage or expiration. Breakage refers to product loss from typical conditions of transportation, packaging, and handling, while expiration refers to product losses that occurred when a product reached the end of its lifespan before being purchased by a consumer or broken (see Sections 6.3.9 and 6.3.11 for more detail).

## 3.3 How vegetables were transported through the supply chain

A vegetable’s path through the supply chain was determined largely by geographic proximity: vegetables from village markets were *transported to* the closest wholesale market, and retailers *retrieved vegetables from* the closest wholesale market. The path from village market to wholesale market to retailer was unidirectional, with products flowing according to the amount of supply entering the system through village markets and wholesale markets.

Geographic areas of Odisha were connected through bidirectional trade at the wholesale level which was triggered on the basis of demand. For example, products at a wholesale market could either proceed to a retailer in close vicinity, or to a wholesale market in another geographic area if the distant wholesale market’s stock fell below a threshold of its typical daily demand.

Not every wholesale market traded with every other wholesale market; the markets traded in the hierarchical structure shown in **Figure 2** in which wholesale markets were classified as Tier 1 (n=4), Tier 2 (n=8) or Tier 3 (n=395) markets, where Tier 1 and Tier 2 markets were higher-volume markets that traded with each other to bridge geographic distances. To identify which wholesale markets were likely to bridge geographic distances, data were used to identify wholesale markets where typical transaction volumes were disproportionately high in relation to nearby population size or estimated agricultural production (see Section 6.1.3 for more detail).

# Supplemental Section 4. Model Runs

## 4.1 Time horizon

HERMES Agrifood was written in Python. A “model run” (or “simulation run”) consists of setting the simulation in motion for a specific time horizon (in this case, 12 months) and recording the model output as a series of .csv files. The time horizon of one simulation year allowed us to assess long-term processes and seasonal fluctuations. One simulation year consisted of 28 days per month and 12 months per year for a total of 336 simulation days. The simulation year was preceded by a “burn-in” period of 7 days to account for the fact that the virtual supply chain begins “empty” and is then populated with products.

## 4.2 Number of iterations per run

Each model run consisted of multiple iterations, with results representing the average of five iterations in order to account for variance between stochastic runs. While some model parameters were fixed (e.g., the distance between two locations), others were stochastic and drew from a probability distribution. For example, the timing of vehicle departures from locations, the timing of consumer arrivals at retail locations, and the rate at which unavoidable product loss occurred during storage and transport drew from Poisson distributions.

The choice of five iterations per scenario was guided by Bryne’s estimate that for stochastic model runs, achieving a precision of results within 5% of the mean requires five runs if the coefficient of variation is below 0.10.^14^ For the model outcomes of interest (e.g., supply ratios and product loss), the coefficient of variation from preliminary model runs was well under 0.10.

## 4.3 Compute resources

High performance compute clusters were used conduct model runs of the system at baseline and under experimental conditions. Computational time varied depending on the number of locations, routes, and products; the time horizon; and the specific nature of processes represented (e.g., how frequently a location calculated the demand of another location it supplies). The model runs described here contained 10,543 distinct locations represented by 21,855 computational entities^[[3]](#footnote-3)^ and connected by 29,377 routes. A typical run (including five iterations with a one-year time horizon) required 12 to 18 real-time hours to complete on a computational node with 128 gigabytes (GB) of random access memory (RAM).

# Supplemental Section 5. Model Outcomes

As a discrete event simulation, HERMES Agrifood tracked the status of every location, device, and product in the system. HERMES Agrifood generated outcomes related to supply, demand, demand fulfillment, resource utilization, supply chain agility, and logistics costs.^15^ Model outcomes could be reported for individual locations or routes, or as aggregate outcomes for supply chain levels or geographic regions. This section describes model outcomes that were assessed in this manuscript; they represent only a subset of possible model outcomes that can be computed.

## 5.1 Outcomes related to product availability

The *supply ratio* is a measure of demand fulfillment. A tomato supply ratio of 80% means that when simulated consumers arrived at supply chain locations (i.e., village markets, wholesale markets, or retailers) throughout the simulation run, tomatoes were available 80% of the time. For the remaining 20% of the time, when simulated consumers arrived at the location the product was not available. Supply ratios are specific to each product and can be reported for individual locations, for supply chain levels, for the supply chain as a whole, or disaggregated by other characteristics (e.g., rural or urban markets).

$$\boldsymbol{Supply ratio}= \frac{\# Instances a product was available when a consumer arrived at a market}{\# Instances consumers arrived at a market to purchaes a product during the simulation run}$$

## 5.2 Outcomes related to product loss

Total product loss was the sum of two types of product loss, each representing a different mechanism of loss: breakage or expiration. A total product loss of 15% for brinjal means that 15% of all brinjal that entered the supply chain was lost due to breakage or expiration during the simulation run.

$$\boldsymbol{Total product loss}= Product expiration+Product breakage$$

$$\boldsymbol{Product expiration}= \frac{Amount of product that expired because it reached its maximum lifespan}{Amount of product that entered the supply chain}$$

$$\boldsymbol{Product breakage}= \frac{\begin{aligned} Amount of product broken during storage or transport due to unavoidable factors \\ \left( e.g., accidental spills \right) and typical conditions of packaging, handling, and transport \end{aligned}}{Amount of product that entered the supply chain}$$

## 5.3 Outcomes related to supply chain performance

Assessing time through the supply chain characterizes the supply chain’s agility.

$$\boldsymbol{Time through the supply chain}= Average \# days a product spends in the supply chain between the time$$

$$\boldsymbol{Time through the supply chain}= it enters the supply chain and the time it is purchased,broken, or expired$$

# Supplemental Section 6. Model Inputs (data sources, parameter values, and assumptions)

The major components of HERMES Agrifood are the supply chain structure, locations, storage device at locations, transportation routes between locations, vehicles, personnel, products, and demand for products. Each component has attributes; for example, attributes of products include packed volume and lifespan at various storage conditions. These attributes influence the way resources flow through the system during simulation runs, as well as logistics costs incurred.

For each component and attribute of the HERMES Agrifood model, this section describes data sources, parameter values, and assumptions. **Table S1** provides a high-level summary of the main data sources used to develop model inputs. Extant data sources included peer-reviewed literature, government publications and databases, technical reports, and spatial data. For each model parameter, a literature search was conducted and sources were selected on the basis of the authoritativeness of the source, rigor of data collection methods, recentness, and relevance to the context of vegetable supply chains in Odisha. Data sources were triangulated against each other, and in some cases, multiple data sources were integrated to inform a model mechanism or a specific set of parameter values. When seeking a single parameter value (e.g., the lifespan of a tomato in cold storage), in some cases values from the literature were averaged, and in other cases a single value from an authoritative, rigorous, recent source was chosen to populate the model. Details are provided below.

Table S1. Overview of main data sources used to develop model inputs

| Organization or author | Year | Title | Type of data provided | Geography (scale) | Section |
| --- | --- | --- | --- | --- | --- |
| Global Administrative Area Maps (GADM)^16^ | 2017 | GADM Database of Global Administrative Areas: India | Spatial data: administrative boundaries and geophysical features | India (national and state boundaries) | 6.1 |
| India Map Store^17^ | 2017 | Map of Odisha block and district boundaries | Spatial data: administrative boundaries | Odisha, India (district and block boundaries) District and block | 6.1 |
| Google Maps^18^ | 2017 | Google Maps Application Programming Interface (API) | Used to produce lists of geo-located village markets and wholesale markets | Geolocations (latitude and longitude coordinates) | 6.1, 6.1.1, 6.1.2 |
| Google Directions^19^ | 2017 | Google Directions Application Programming Interface (API) | Used to produce driving distances | Driving distances in kilometers | 6.2.3 |
| Government of India, Ministry of Home Affairs, India Census Bureau^20^ | 2011 | India 2011 Demographic Census: District Census Handbook for Odisha | Identifies names of 5,884 villages that have village markets (weekly *haats* or daily *mandis)* | Odisha, India (specific villages) | 6.1.1, 6.1.5, 6.1.6,  6.2.3,  6.3.2,  6.3.3 |
| Government of India, Ministry of Agriculture & Farmers Welfare, Directorate of Marketing and Inspection (DMI)^21^ | 2004 | Directory of Wholesale Agricultural Produce Assembling Markets in India | Identifies names of 405 villages or municipalities that have wholesale markets in Odisha | Odisha, India (specific villages or municipalities) | 6.1.2, |
| Government of India, Ministry of Agriculture & Farmers Welfare, Directorate of Marketing and Inspection (DMI)^22^ | 2017 | AgMarknet | Voluntary online reporting system for wholesale markets; as of 2017, included crop transaction volumes for 106 wholesale markets in Odisha | Odisha, India (specific wholesale markets) | 6.1.2, 6.1.3,  6.1.4, 6.1.7,  6.3.4 |
| Columbia University, Center for International Earth Science Information Network (CIESIN)^23^ | 2016 | Gridded Population of the World dataset, Version 4: Population Count | Population counts for each square kilometer of land | Odisha, India (square kilometer) | 6.1.3, 6.1.5,  6.4.3 |
| Srivastava in *International Journal of Retail & Distribution Management*^24^ | 2008 | Changing retail scene in India | Estimates of retail density (all goods) | India (national) | 6.1.5 |
| Government of Odisha, Department of Agriculture and Farmers Empowerment, Orissa Horticulture Development Society (OHDS)^25^ | 2012 | Project Report on 10MT Cool Chamber | Specifications for cold storage devices | India (national) | 6.1.9 |
| National Centre for Cold Chain Development (NCCD)^26^ | 2015 | All India Cold Chain Infrastructure Capacity Assessment of Status and Gap | Specifications for cold storage devices | India (national) | 6.1.9 |
| National Centre for Cold Chain Development (NCCD)^27^ | 2016 | Cold Chain Development for Fruits and Vegetables in India: Kinnow Cold Chain Study | Specifications for cold storage devices | India (national) | 6.1.9 |
| Winrock International for the United States Agency for International Development (USAID)^28^ | 2009 | Empowering Agriculture: Energy Options for Horticulture | Specifications for cold storage devices | Global | 6.1.9 |
| Kitinoja and Thompson in *Stewart Postharvest Review*^29^ | 2010 | Pre-cooling systems for small-scale producers | Specifications for cold storage devices | Global | 6.1.9 |
| Wilbur Smith Associates, prepared for the Government of India, Ministry of Urban Development^30^ | 2008 | Study on Traffic and Transportation Policies and Strategies in Urban Areas in India | Typical road speeds | India (national, urban areas) | 6.2.4 |
| Alam and Ahmed in *Transport and Communications Bulletin for Asia and the Pacific^31^* | 2013 | Urban transport systems and congestion: a case study of Indian cities | Typical road speeds | India (national, urban areas) | 6.2.4 |
| Pal and Roy in *Transportation in Developing Economies^32^* | 2016 | Impact of Roadside Friction on Travel Speed and LOS of Rural Highways in India | Typical road speeds | India (national, rural areas) | 6.2.4 |
| Government of India, Ministry of Road Transport & Highways^33^ | 2015 | Road Transport Year Book (2013-2014 and 2014-2015) | Types of vehicles used | India (national) | 6.2.5 |
| TrucksDekho^34^ | 2016 | Vehicle Specifications | Vehicle specifications including cargo capacity | India (national) | 6.2.6 |
| Government of India, Agriculture Census Division^35^ | 2011 | Agriculture Census, Table 6B: Estimated Irrigated and Unirrigated Area by Size Classes Under Crop, Tehsil Tables | Vegetable-specific crop production (hectares under production for any given vegetable per block) | Odisha, India (block level, n=315) | 6.1.3,  6.3.1, 6.3.2,  6.3.3,  6.3.5,  6.3.6 |
| Government of India, Ministry of Agriculture and Farmers Welfare, National Horticulture Board (NHB)^36^ | 2012 | Horticulture Crops Estimate for the Year 2011-12 | Vegetable-specific crop production (hectares under production and annual tons produced) | Odisha, India (district level, n=30) | 6.3.1 |
| Government of India, Ministry of Agriculture and Farmers Welfare, National Horticulture Board (NHB)^37^ | 2015 | Yield Comparison of Horticulture Crops State Wise | Vegetable-specific crop yields (tons per hectare), used to convert hectares under production to tons produced | Odisha, India (state) | 6.3.1 |
| Indian Agricultural Statistics Research Institute (ICAR) ^38,39^ | 2003 | Applications of Remote Sensing and GIS in Agricultural Surveys (Module 1.6: Agriculture Census and Module 17: Horticulture Surveys | Information about sampling strategies in the Agriculture Census | India (national) | 6.3.1 |
| Government of India, Ministry of Agriculture and Farmers Welfare, National Horticulture Board (NHB)^40^ | 2014 | Indian Horticulture Database | Classification of each calendar month as a peak, lean, or off production season for specific horticultural crops | Odisha, India (state) | 6.3.4 |
| Sharan and Rawale for the Indian Institute of Management, Ahmedabad^41^ | 2003 | Physical Characteristics of Some Vegetables Grown in Ahmedabad Region | Bulk densities for specific horticultural crops | Ahmedabad, India (applied estimates from another state to Odisha) | 6.2.6,  6.3.7 |
| Multiple sources were used to inform vegetable lifespans, including government sources^42,43^ and peer-reviewed literature^44-53^ | 2004-2017 | Multiple sources | Average lifespans in ambient or cold storage conditions for each vegetable (multiple estimates were compared to produce these averages) | Estimates were drawn from studies based in Odisha,^44^ India (other states or at a national scale),^42,43,45-48^ Bangladesh,^49^ China,^50^ Ghana,^51^ Nigeria,^52^ and the Balkans^53^ | 6.3.9,  6.3.10,  6.3.11 |
| Multiple sources were used to inform average rates of postharvest loss at various supply chain stages, including government sources^13,54,55^ and peer-reviewed literature^4,6,56-58^ |  | Multiple sources | Average rates of postharvest loss at various supply chain stages (multiple estimates were compared to produce these averages rates) | Estimates were drawn from studies based in Odisha,^6^ India (other states or at a national scale), ^4,13,54-56,58^ and in low- and middle-income countries generally^57^ | 6.3.11 |
| Government of India, Ministry of Statistics and Program Implementation, National Sample Survey Office^59^ | 2010 | Household Consumption Expenditure, NSSO 68^th^ Round | Annual per capita expenditures per kg of specific food commodities | Odisha, India (state, with rural and urban estimates provided separately) | 6.1.5,  6.3.5,  6.3.6,  6.4.2 |

## 6.1 Locations and Storage Devices

Maps presented here were created in QGIS.^60^ Spatial data for India national and state boundaries and geophysical features (e.g., water, railways, roadways) were from the Global Administrative Areas dataset.^16^ Spatial data for Odisha administrative boundaries at the district and block level were from the India Map Store.^17^ Specific geolocations (i.e., latitude and longitude coordinates) were obtained from the Google Maps Application Programming Interface (API)^18^ using the World Geodetic System (WGS) 84 global reference system.

### 6.1.1 Locations of Village Markets

**Figure S2** shows the locations of village markets included in the model. Village market geolocations were identified as the geographical center of any village indicated in India’s 2011 Demographic Census as having a weekly *haat* or daily *mandi* (local terms for different types of markets. ^20^ The census identified 5,884 villages in Odisha with weekly *haats* or daily *mandis*, which constitutes 11.5% of the total 51,313 villages in Odisha. The model included village markets in 5,449 villages for which accurate geolocations could be obtained (92.6% of the 5,884 indicated in the census). The 7.4% of excluded locations were those for which a village name in the demographic census did not match a village name within the boundaries of the state of Odisha using the Google Maps API; villages with duplicate village, block, and district names but different geolocations; and villages with duplicate geolocations but different names.


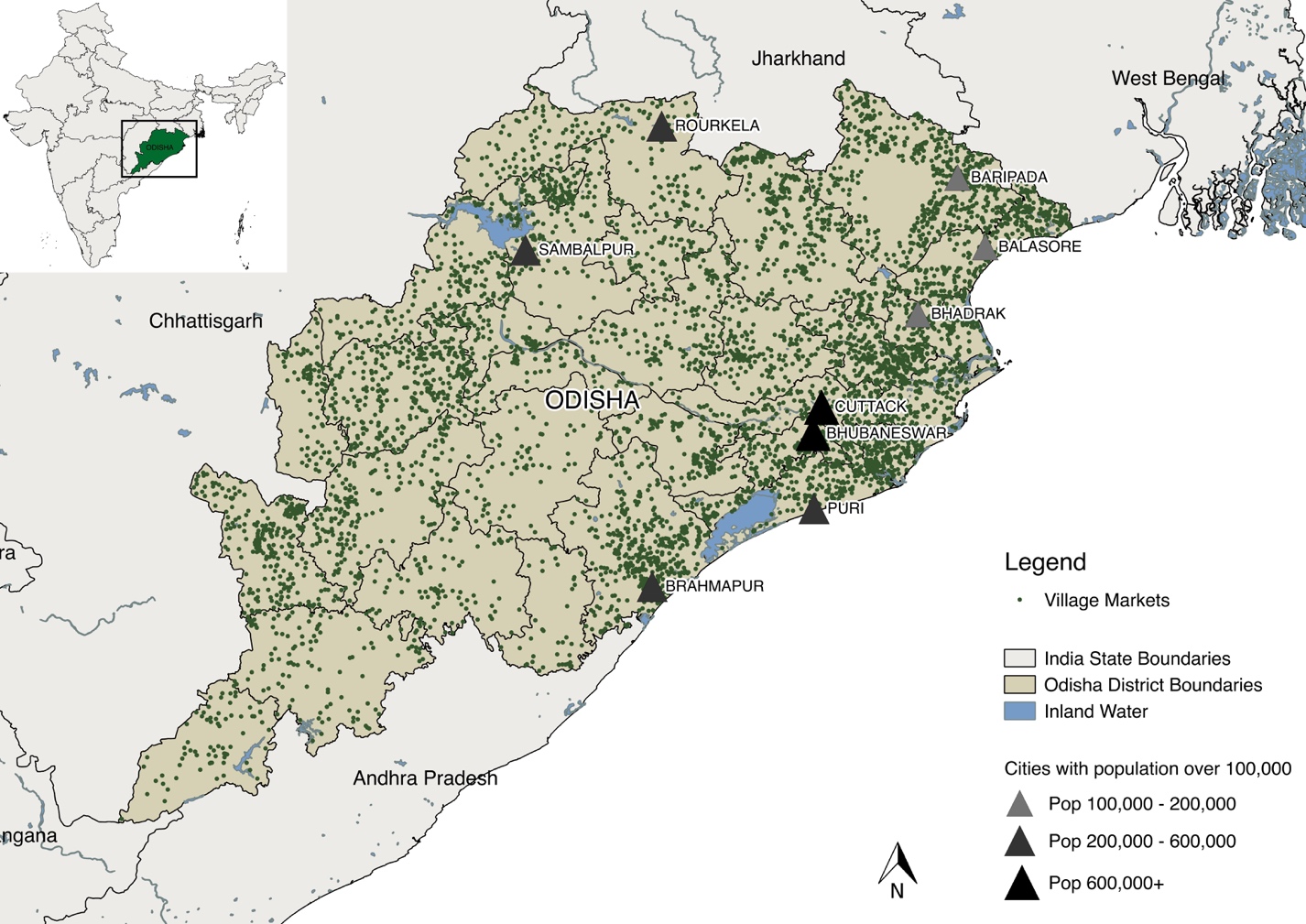


Figure S2 Locations of village markets in the model.

The model included 5,449 village markets, with locations informed by the Demographic Census.

### 6.1.2 Locations of Wholesale Markets

**Figure S3** shows the locations of wholesale markets included in the model. A directory of wholesale agricultural produce markets from the Government of India’s Directorate of Marketing and Inspection identified the locations (i.e., the village or municipality) of 405 wholesale markets.^21^ Wholesale market locations were defined as the geographic center of the village or municipality, and the Google Maps API was used to obtain geolocations. The wholesale directory was triangulated with AgMarknet, an online reporting system for wholesale markets operated by the government of India’s Directorate of Marketing and Inspection.^22^ All of the 106 wholesale markets in Odisha that appear in the AgMarknet reporting system also appear in the wholesale directory.


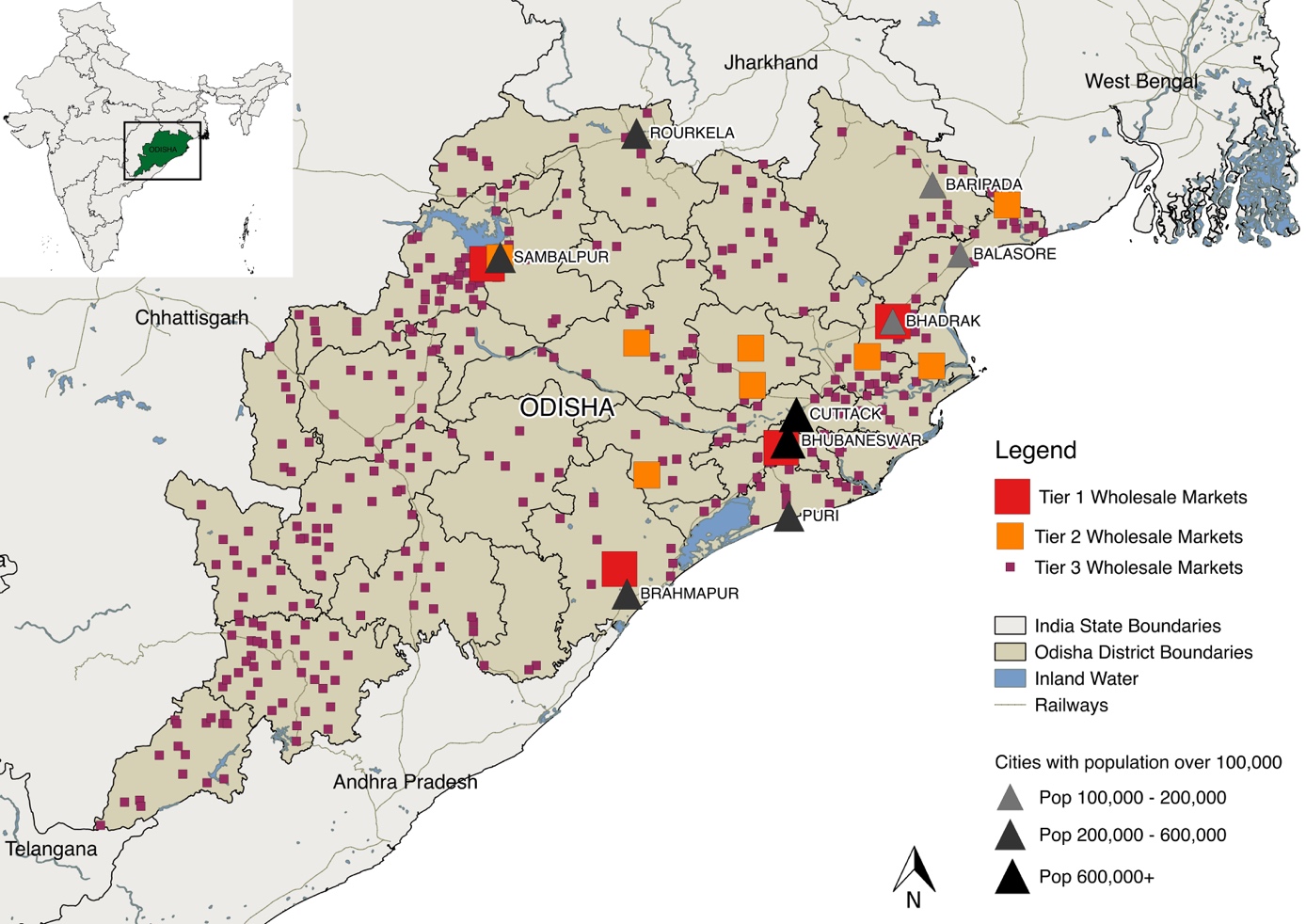


Figure S3 Locations of wholesale markets int he model.

The model included 405 wholesale markets, with locations informed by the Government of India’s Directorate of Marketing and Inspection. The wholesale markets traded in a hierarchical network: all Tier 1 markets (n=4) traded with each other, to connect geographic areas of the state. Tier 2 markets (n=8) traded with both Tier 1 and Tier 3 markets, to aggregate products regionally. Tier 3 (n=383) markets only traded with a single other wholesale market (the closest Tier 1 or Tier 2 market).

### 6.1.3 Creating networks of bidirectional trade between wholesale markets

The supply chain included bidirectional trade because wholesale markets traded with each other. Not every wholesale market traded with every other wholesale market; the markets traded in the hierarchical structure shown in **Figure S3** in which wholesale markets were classified as Tier 1, Tier 2 or Tier 3 markets. Tier 1 and Tier 2 markets were higher-volume markets that traded with each other to bridge geographic distances. Tier 3 markets were lower-volume markets that traded only with the closest Tier 1 or Tier 2 market. To identify which wholesale markets were likely to bridge geographic distances, we identified wholesale markets where typical transaction volumes were disproportionately high in relation to nearby population size or estimated agricultural production. **Figure S4** shows two scatterplots used to make these comparisons.

At the wholesale markets labeled in the scatterplot on the left side of Figure S5, the amount of wholesale vegetable transactions outpaced the population served. 2017 wholesale market crop transaction volumes from the Government of India’s Directorate of Marketing and Inspection^22^ (see **Figure S5**) were compared with the estimated catchment area of each wholesale market (i.e., the number of people for whom a specific wholesale market is closer than any other). To estimate population catchment areas, wholesale market geolocations were combined with population counts from the Columbia University Gridded Population of the World dataset (see **Figure S6**).^23^

At the wholesale markets labeled in the scatterplot on the right side of Figure S5, the amount of wholesale vegetable transactions outpaced nearby vegetable production. Wholesale market transaction volumes were compared to the estimated amount of vegetable production that would be expected in areas near the wholesale market. To estimate vegetable production per wholesale market, block-level vegetable production in tons from the Government of India Agriculture Census Division^35^ (see **Figure S13**) were divided evenly among the number of wholesale markets per block.

Wholesale markets that were identified through both methods–comparing wholesale transactions to population served, and comparing wholesale transactions to nearby production–were selected as Tier 1 or Tier 2 markets. The Tier 1 markets–Aiginia, Bhadrak, Gosala, and Hinjilicut–not only have high transaction volumes, but are located in different geographic areas of Odisha. The hierarchical network of wholesale markets is described in **Table 2** and shown geospatially in **Figure S3.**

Figure S4 Comparison of wholesale market vegetable arrivals to nearby population size and vegetable production

These comparisons were used to identify which wholesale markets served as key markets to bridge geographic areas.

Table S2. Types of wholesale markets in the hierarchical network of lateral trade

| Wholesale market type | Number included | Trading partners | Function |
| --- | --- | --- | --- |
| Tier 1 | 4 | Trades with all other Tier 1 markets, and any Tier 3 markets that feed into it | Connect geographic areas of the state |
| Tier 2 | 8 | Trades with the closest Tier 1 market, and any Tier 3 market that feed into it | Regional aggregation of products to Tier 1 markets |
| Tier 3 | 395 | Only trades with the closest Tier 1 or Tier 2 market | Pull products from Tier 1 and 2 markets to meet needs of nearby retailers, and provide surplus production to Tier 1 and 2 markets |


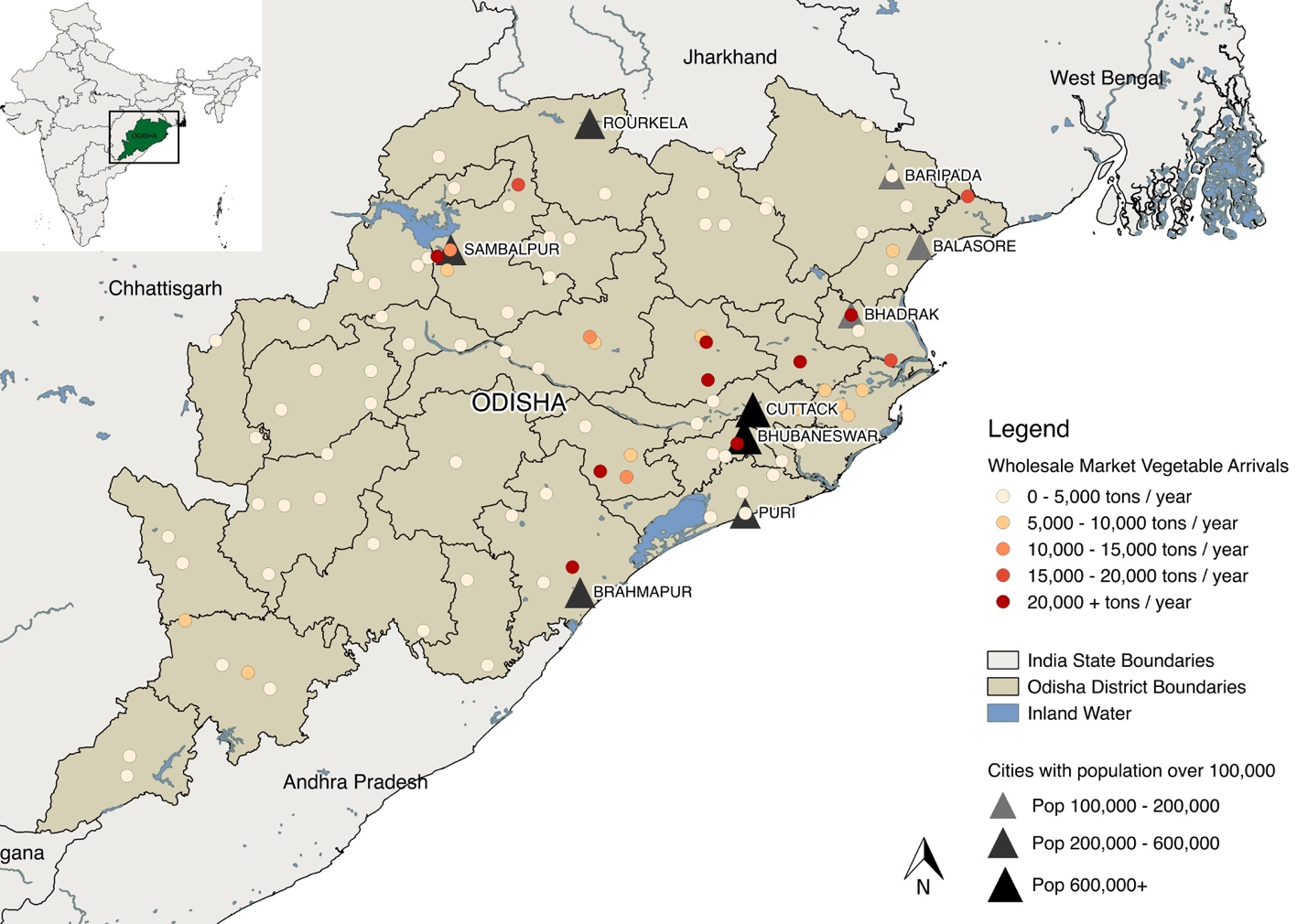


Figure S5 Amount of vegetables (potato, onion, tomato, brinjal, and cabbage), traded at wholesale markets within the AgMarknet system.

Of the 405 wholesale markets included in the model, 106 wholesale markets reported data on food commodity arrivals and prices through the AgMarknet system in 2017. This map shows the locations of those 106 wholesale markets and indicates the total tons of vegetables arrivals (potato, onion, tomato, brinjal, and cabbage) they reported in 2017. These data on wholesale arrivals were used to help determine which markets were most likely to serve as key markets that bridge geographic distances (Tier 1 and Tier 2 wholesale markets in the model).

### 6.1.4 Validating networks of bidirectional trade between wholesale markets

To validate whether the network of bidirectional trade distributed vegetables throughout Odisha’s supply chain in a way that reflects the real world, we compared the amount of vegetables passing through modeled wholesale markets from a baseline model run to empirical data on wholesale vegetable transactions.

The empirical data were from the Directorate of Marketing and Inspection’s AgMarknet reporting system. The AgMarknet system included 106 wholesale markets in Odisha, of which 95 markets reported data on weekly arrivals (in tons) of potato, onion, tomato, brinjal, or cabbage in the year 2017.^22^ Data from these 95 markets were compared to baseline results from the subset of 95 matching wholesale markets in the model. In the modeled results, the movement of products throughout the supply chain was driven by production amounts, geographic distribution, and timing from the Agriculture Census and National Horticulture Board. Although the AgMarknet data were used to identify which of the state’s markets would serve as Tier 1 or Tier 2 wholesale markets, the actual wholesale arrival amounts were not used as a model input, and thus were an appropriate comparison for validation.

The scatterplot in **Figure S6** compares data on real-world wholesale market vegetable transactions to modeled results under baseline conditions for the full set of markets for which AgMarknet data were available (95 of 106 wholesale markets). The Pearson correlation between real-world and modeled wholesale market transactions was 0.89. In an analysis with outliers dropped (any markets where modeled wholesale market arrivals were more than 3 standard deviation above or below the mean, which excluded 3 to 4 markets depending on the vegetable), the Pearson correlation remained robust at 0.81 (not shown).


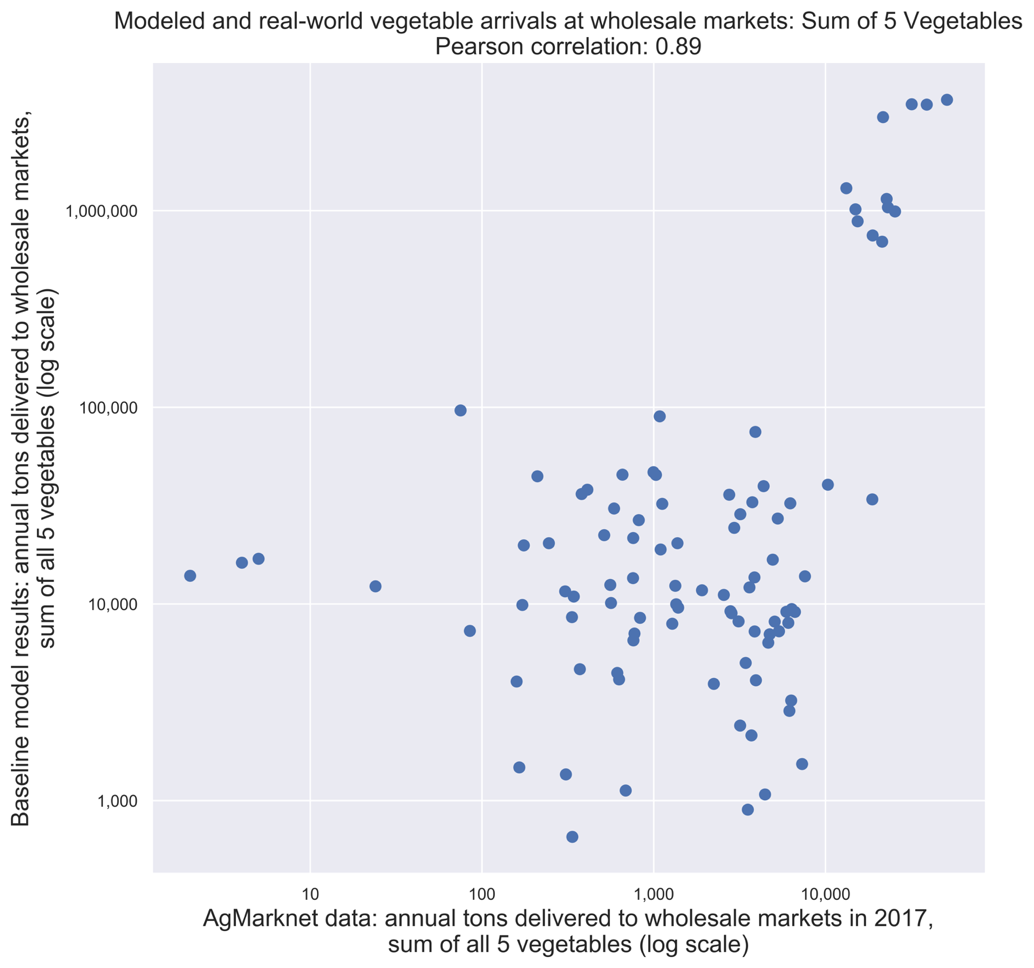


Figure S6 Modeled and real-world vegetable arrivals and wholesale markets: Sum of 5 vegetables, shown on a log scale.

This scatterplot compare real-world data on vegetable arrivals (sum of potato, onion, tomato, brinjal, and cabbage) at Odisha wholesale markets (X-axis) to modeled results from the system at baseline (Y-axis). Data are shown on a log scale.

The scatterplots in **Figure S7** show a similar comparison, but for each individual vegetable rather than the sum of all five. Pearson correlations between modeled and real-world wholesale data for individual vegetables were moderately strong, ranging from 0.40 to 0.80, and remaining moderately strong when excluding outliers (0.29 to 0.82, not shown). For ease of visual interpretation, Figure S7 displays data on a log scale and Figure 8 displays data on a linear scale.

**
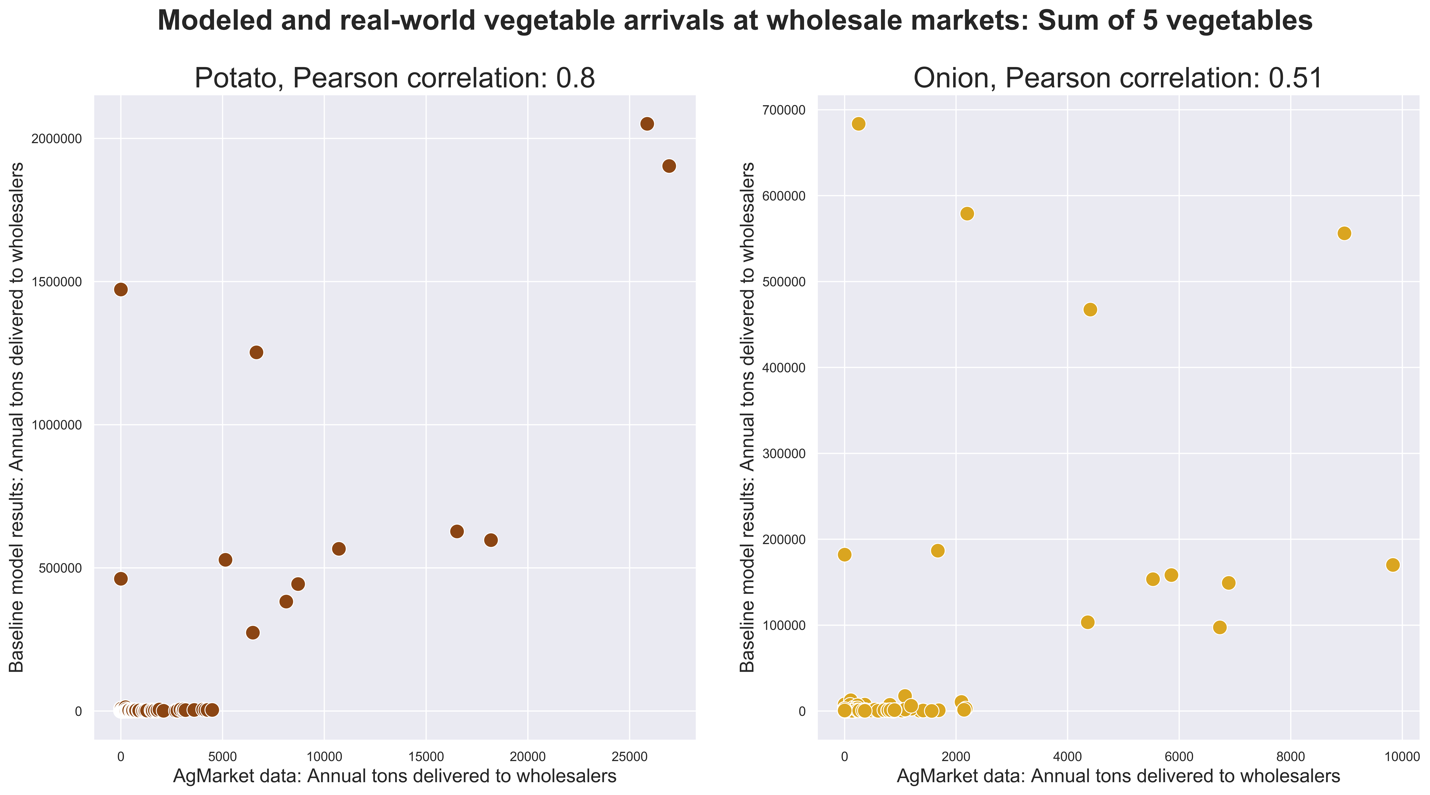
**

**
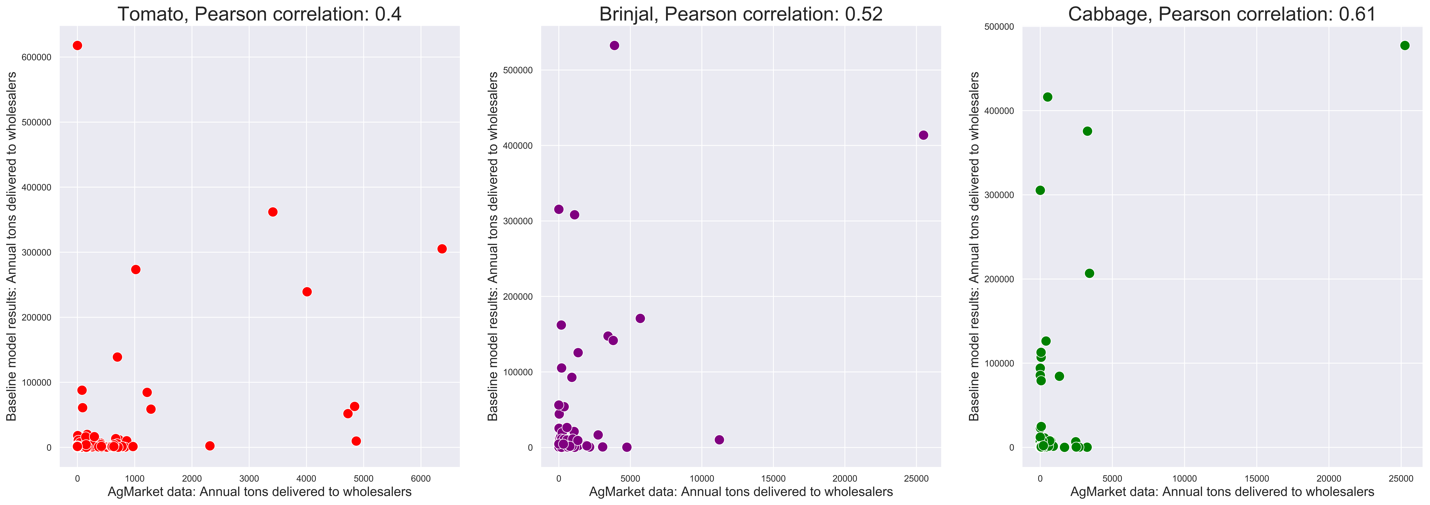
**

Figure S7 Modeled and real-world vegetable arrivals at wholesale markets.

This scatterplot compare real-world data on vegetable arrivals at Odisha wholesale markets (X-axis) to modeled results from the system at baseline (Y-axis) for five individual vegetables. Data are shown on a linear scale.

It should be noted that the empirical data on vegetables arrivals from AgMarknet reflect smaller amounts of wholesale transactions (up to ~50,000 tons of total vegetables annually at the largest market) than the modeled baseline data (up to ~3.5 million tons of total vegetables annually at the largest market). This is due to the fact that the AgMarknet data are reported by wholesale markets on a voluntary basis, and so the AgMarket data would be expected to under-estimate real transactions. Nonetheless, the AgMarket data provide a useful indicator of the relative magnitude of transactions between wholesale markets.

Overall, these correlation coefficients indicate that the modeled movement of vegetable through Odisha’s supply chain reflects the real-world transactions at wholesale markets fairly well—even when dropping outliers (particularly high-volume markets), and whether we examine total vegetables or individual vegetables—lending validity to the modeled distribution of products throughout the system.

### 6.1.5 Locations of Retailers

Because vegetable retail in India occurs primarily through the informal sector, the locations of specific retailers in Odisha are generally not known. In the model, specific retail locations were generated and assigned based on densities that differed between urban, peri-urban, and rural areas of Odisha.

To define each square kilometer of within Odisha as urban, peri-urban, or rural, guidelines were applied to spatial population count data in order to match the demographic composition reported India’s 2011 Demographic Census. The 2011 Demographic Census reported that 17% of Odisha’s population lived in urban areas, with the remaining 83% in rural areas.^20^ The demographic census did not report on peri-urban populations, and it also did not provide spatial data. Spatial data on population counts were available through the Gridded Population of the World dataset, which shows population counts for every square kilometer of the earth’s surface, as shown in **Figure S8.**^23^ To categorize each square kilometer within Odisha as urban-, peri-urban, or rural, the following guidelines were used: each square kilometer with a population density over 700 people per square kilometer was classified as urban, each square kilometer within a 5 kilometer boundaries of an urban areas was classified as peri-urban, and all remaining square kilometers were classified as rural. A minimum population density of 700 people per square kilometer was chosen for urban areas because this yielded a 17% urban population, which matched the demographic census report. **Table S3** summarizes these guidelines and the population statistics that they produced, and **Figure S9** shows the urban, peri-urban, and rural boundaries spatially.


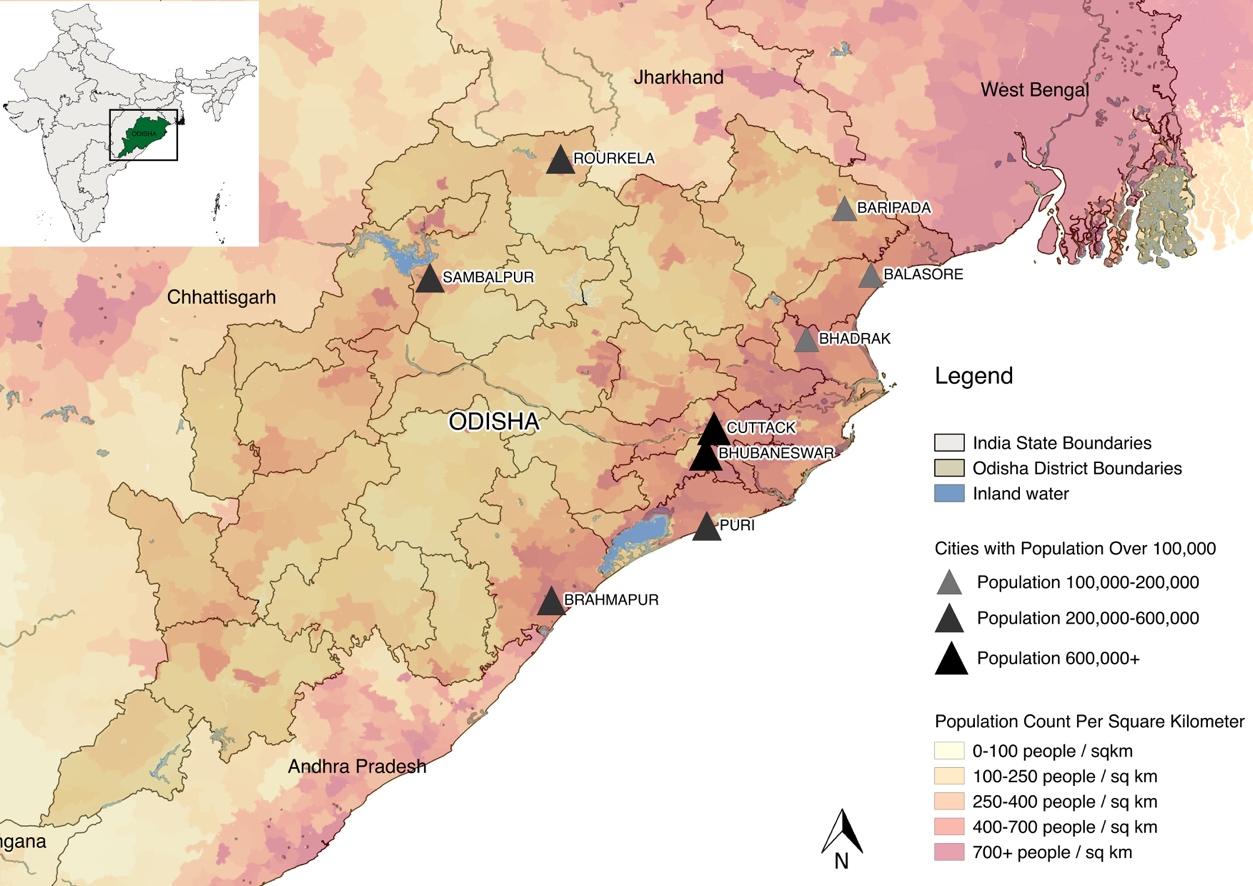


Figure S8 Population counts per square kilometer in Odisha.

This map shows data from the Gridded Population of the World dataset. The population counts per square kilometer were used to help identify areas within Odisha as urban, peri-urban, or rural, in combination with the guidelines shown in Table S3.

Table S3. Characteristics of areas classified as urban, peri-urban, and rural

| Urbanicity class | Guidelines used to define each urbanicity class | Percent of Odisha’s total population captured by each urbanicity class | Average population density in each urbanicity class |
| --- | --- | --- | --- |
| Urban | Areas with population density over 700 people / km^2^ | 17% | 1349 people / km^2^ |
| Peri-urban | Areas within a 5km buffer around the outer boundary of urban areas | 9.6% | 429 people / km^2^ |
| Rural | Aras outside the urban or peri-urban boundaries | 73.3% | 184 people / km^2^ |


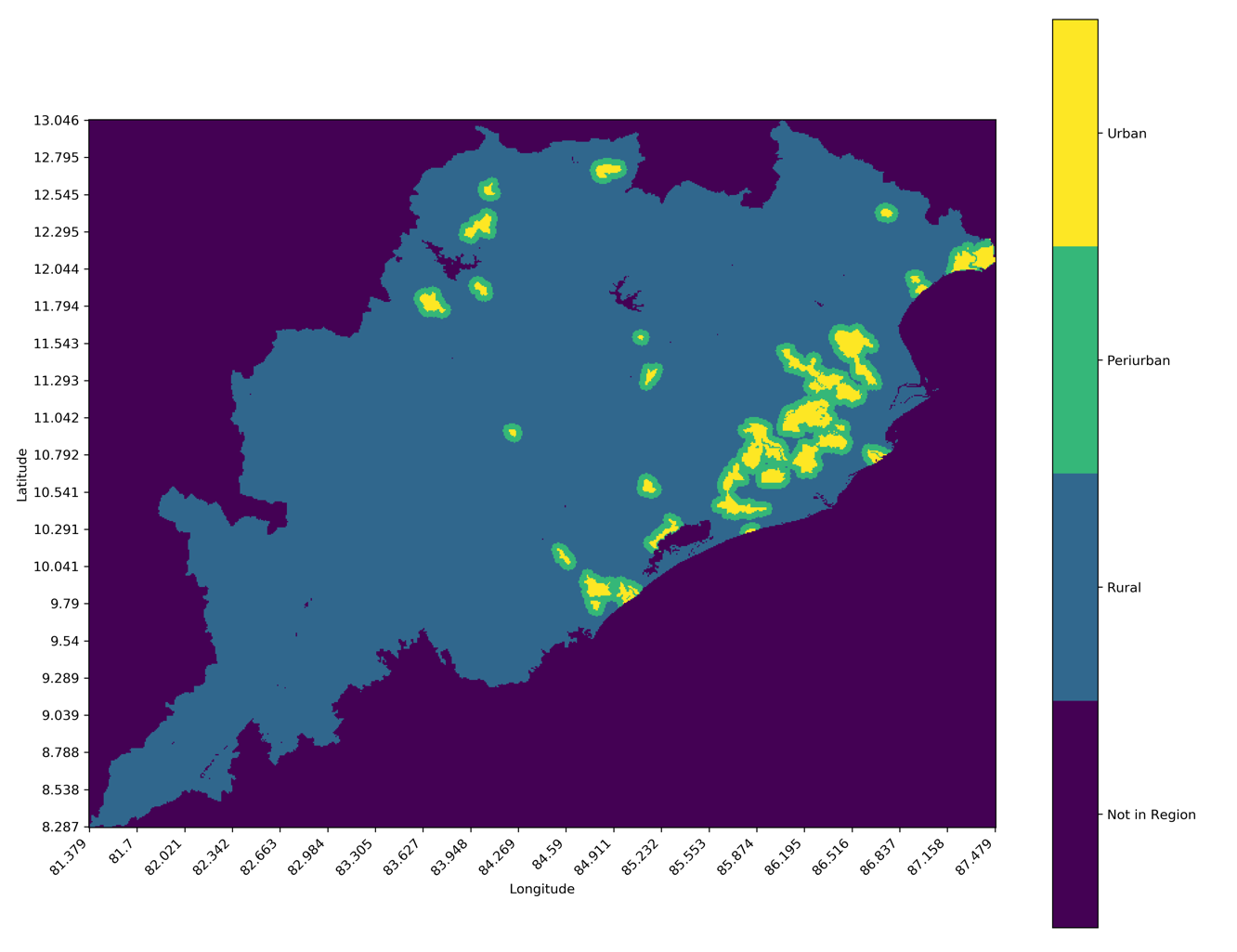


Figure S9 Map of area classified as urban, peri-urban, and rural in the model.

These urban, peri-urban, and rural boundaries were created using the guidelines in Table S3.

Once all spatial areas within Odisha were classified by urbanicity, the specific locations of retailers were assigned geolocations based on the following guidelines: in urban and peri-urban areas 1 retailer was assigned for every 350 people, and in rural areas one retailer was assigned for every 400 people. **Figure S10** displays retailer locations spatially and **Table S4** summarizes the characteristics of retailers in urban, peri-urban, and rural areas.

**Figure S11** details the distribution of distances between retailers and wholesale markets by urbanicity, showing that on average urban retailers were the closest to their nearest wholesale market (mean = 16.5 minutes, SD = 9.1 minutes) and rural retailers were the farthest from their nearest wholesale market (mean = 33.4 minutes, SD = 17.4 minutes).

The choice of retail density was informed by the following: India has approximately one retail shop (for all goods) per 100 people^24^; according to National Sample Survey Office (NSSO) consumer expenditure data, approximately half of household expenditures in India go towards food rather than non-food goods (52.9% in urban areas, and 42.6% in rural areas)^59^; and approximately half of food transactions at wholesale markets are vegetables rather than other foods.^22^ These data would suggest an average retail density of one retailer per 400 people. Other sources in the literature were used to triangulate these data. For example, surveys of urban consumers in India have found that consumers in Chennai walked an average of 0.5km to vegetable retailers^61^ and that consumers in Delhi walked an average of 17 minutes to vegetable retailers.^62^ Surveys of rural consumers in Odisha have found that consumers lived an average of 4.5 km from the nearest vegetable market.^63^


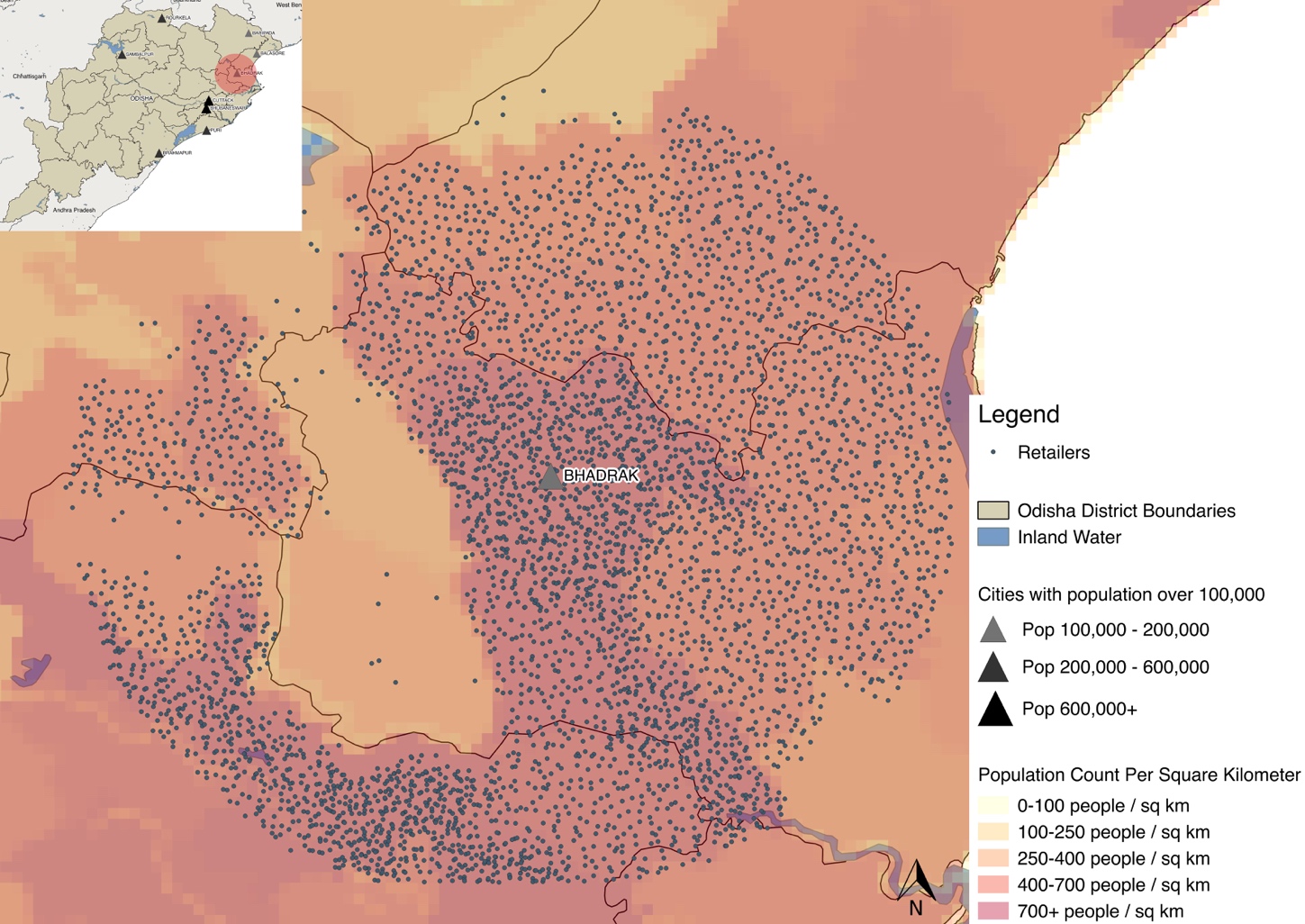


Figure S10 Map of retailers in the focus region.

Though the model represented individual village markets and wholesale markets throughout the state of Odisha, individual locations for retailers were only represented within the “focus region” shown here.

Table S4. Characteristics of retailers in focus region

| Urbanicity class | One retailer was created for every X residents | Number of Retailers in focus region (% of total) | Number of people served by each retailer | Mean distance to closest wholesale market, in driving minutes (SD) |
| --- | --- | --- | --- | --- |
| Urban | 350 | 1943 (41.4%) | 412 | 16.5 (9.1) |
| Peri-urban | 350 | 921 (19.6%) | 623 | 21.5 (12.3) |
| Rural | 400 | 1825 (38.9%) | 645 | 33.4 (17.4) |


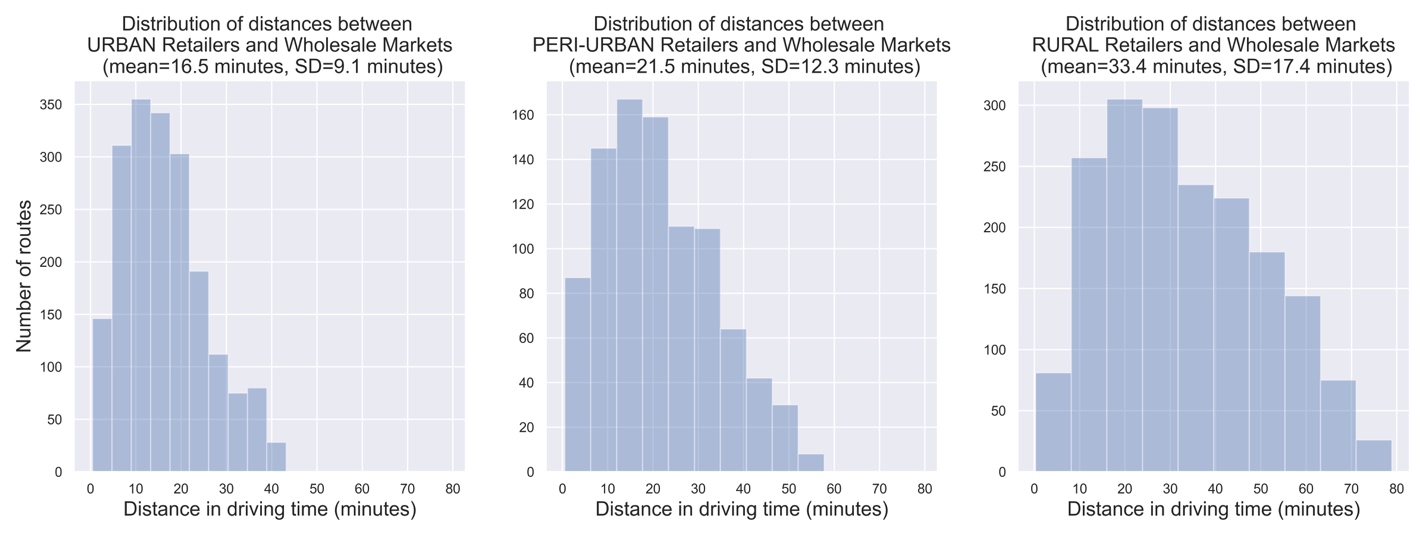


Figure S11 Distribution of distances from retailers to the closest wholesale market in urban, peri-urban, and retail areas.

Retailers received vegetables from the closest wholesale market. The travel time to the closest wholesale market varied by urbanicity, with urban retailers being the closest to wholesale markets and rural retailers being the farthest from wholesale markets on average. These calculated travel times all assume a detour index of 1.425 and an average road speed of 30 kilometers per hour, and they are based on the geolocations described in other sections.

### 6.1.6 Operating schedules at each type of location

One simulation year in HERMES Agrifood consisted of 336 simulation days: 7 days per week, 4 weeks per month, and 12 months per year. At the village market supply chain level, village markets identified in the demographic census as a daily *mandi* operated 7 days per week (30.7% of all village markets), and those identified as a weekly *haat* operated one day per week (69.3% of all village markets).^20^ At all other supply chain levels, transactions took place on a daily basis.

Within any operating market day, transactions took place throughout the day according to the schedule shown in **Figure 3.** The times shown in the figure represent the average time a vehicle would depart a location or a consumer would arrive at a location, with individual times drawing from a Poisson distribution.

### 6.1.7 Vegetables handled at each type of location

All supply chain locations handled all five vegetables. Although wholesale markets in India are sometimes classified as specializing in “green vegetables” (i.e., more perishable vegetables such as tomato, brinjal, and cabbage) or “non-green vegetables” (i.e., less perishable vegetables such as potato and onion), in this model each wholesale market handled all five products. An analysis of the composition of products arriving at the 106 Odisha-based wholesale markets in the AgMarknet reporting system, shown in **Figure S12,** revealed that while some markets predominantly transacted green vegetables or non-green vegetables, 55% of the 106 markets received at least some amount of all five products during the year 2017, and 76% of the 106 markets received at least some amount of four products during the year 2017.^22^


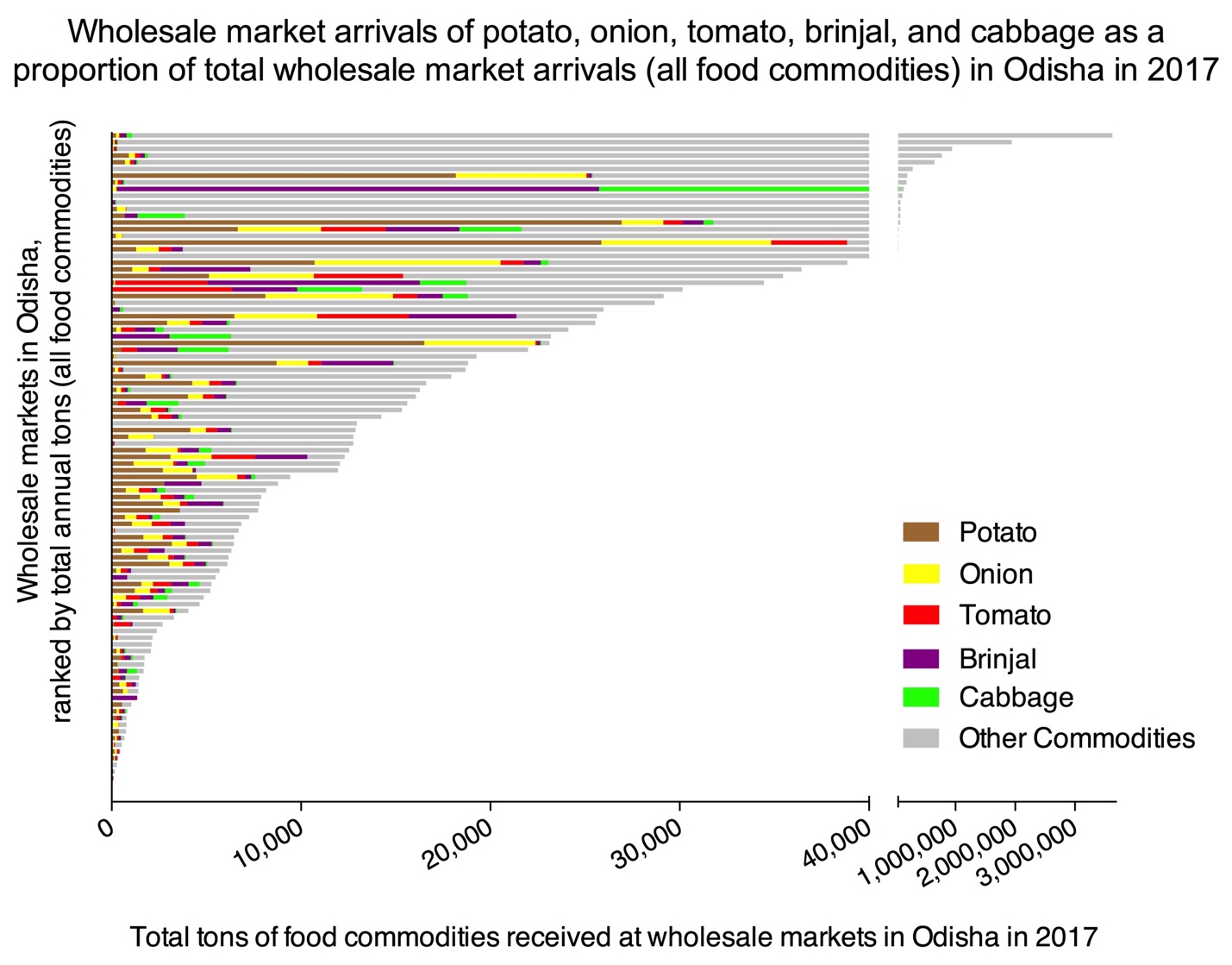


Figure S12 Wholesale market arrivals of potato, onion, tomato, brinjal, and cabbage in the context of total arrivals (all food commodities) in Odisha in 2017, per AgMarknet data.

Each bar represents the composition of vegetable arrivals at a single wholesale market. Note that “Other Commodities” includes rice, pulses, fruits, spices, oils, live animals, animal products, and some non-food commodities (e.g., tobacco, jute, wood).

## 6.2 Routes and Vehicles

### 6.2.1 Order of stops along a route

As products proceeded from one supply chain level to the next, subsequent locations were chosen on the basis of proximity. For example, from any given village market, a product destined for a wholesale market would proceed to the wholesale market with the shortest travel distance.

### 6.2.2 Ordering policies

HERMES Agrifood uses four characteristics to define ordering policies:

1. whether the *quantity* of product shipped is fixed or variable,
2. whether the *schedule* of shipments is fixed or variable,
3. whether the supplier *delivers* the product or the recipients *fetches* the product, and
4. whether the vehicle completes a *single trip or multiple trips* as needed.

**Table S5** describes the ordering policies for each route type in the model. For routes between village markets and wholesale markets, the demand at the wholesale market level was so large that all vegetables (except those purchased by local consumers at the village market level) would proceed to the next supply chain level. Wholesale markets traded with each other on the basis of demand. For trade between Tier 1 or 2 and Tier 3 markets, Tier 3 markets would receive what they needed based on the size of demand from their nearby retailers, and they would provide any surplus to Tier 1 or 2 markets. For trade among Tier 1 and Tier 2 markets, for any given trading partnership, the market with a lower stock would demand half the difference in their stock. For routes between wholesale markets and retail markets, retailers would demand the amount required by their consumers.

Table S5. Description of ordering policies

| Category | Route Description |
| --- | --- |
| Routes between village markets and wholesale markets | 1. Product quantity is variable (depends on amount of production and calculated demand from wholesale markets) 2. Shipment scheduled is fixed (daily or weekly, depending on operating schedule of the village market) 3. Recipient (wholesale market) fetches product from the supplier (village market) 4. Vehicles completes multiple trips as needed |
| Routes between wholesale markets (bidirectional, lateral trade) | 1. Product quantity is variable (depends on calculated demand from wholesale markets and retailers supplied by the origin wholesale market) 2. Shipment scheduled is variable (only as needed) 3. Recipient wholesale market fetches product from supplying wholesale market 4. Vehicle completes multiple trips as needed |
| Routes between wholesale markets and retailers | 1. Product quantity is variable (depends on calculated demand at the Retail level) 2. Shipment schedule is fixed (daily) 3. Recipient (retailer) fetches the product from the supplier (wholesale market) 4. Vehicle completes a single trip |

### 6.2.3 Travel distance

The travel distance between any two locations was calculated as the Haversine distance (i.e., distance around a sphere, commonly used to for navigational distances) multiplied by an average detour index of 1.425. Detour index is the ratio of driving distance to Haversine distance.

To calculate a detour index specific to Odisha, two random lists of 600 villages within Odisha were generated, with villages drawn from the list of geo-located village markets from the India 2011 Demographic Densus.^20^ For 600 routes between locations on these two lists, Haversine distances were calculated from latitude and longitude coordinates using the WGS84 global reference system and driving distances were obtained from Google Maps using the Google Directions API,^19^ and the detour index was calculated as the ratio between the two.

### 6.2.4 Travel time

The travel time between any two locations was calculated as the travel distance multiplied by an average road speed of 30 kilometers per hour. This conservatively low speed takes into account traffic congestion in urban areas, non-paved roads in rural areas, overloaded vehicles, and breaks for drivers. An assessment of urban road speed in India commissioned by the Government of India’s Ministry of Urban Development found road speeds in urban areas to range from 16-20 kilometers per hour.^30,31^ An assessment of road speed on rural highways in India estimated average speed in market areas to be 25 kilometers per hour, with average speeds outside of market areas as 50 kilometers per hour.^32^ **Figure S13** shows distributions of travel times by route type.

| 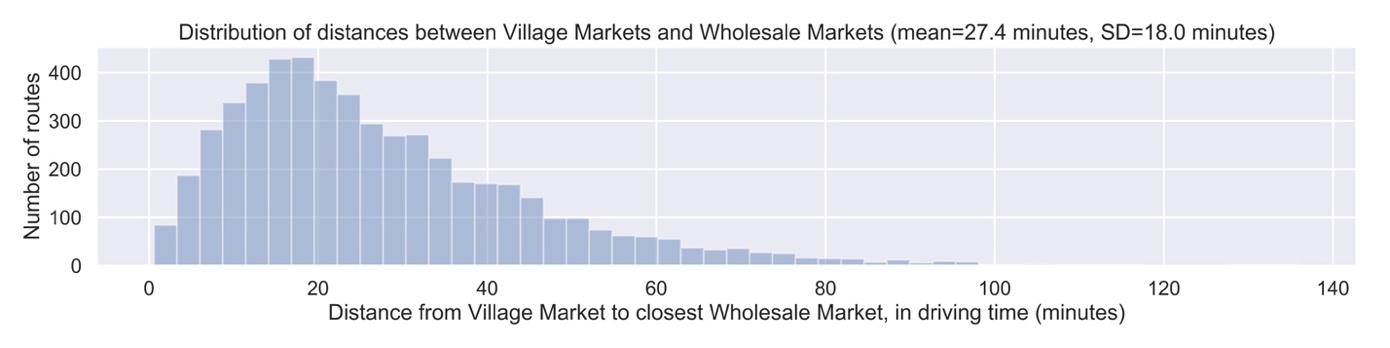 |
| --- |
| 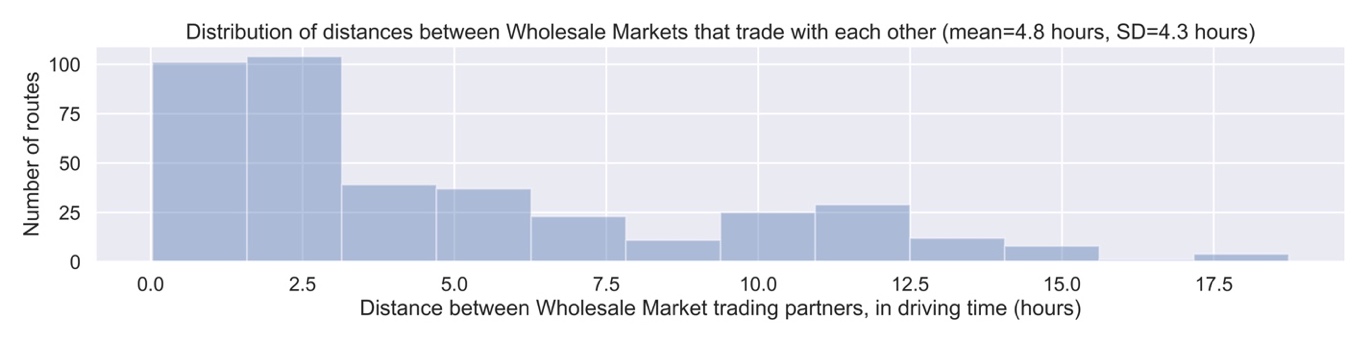 |
| 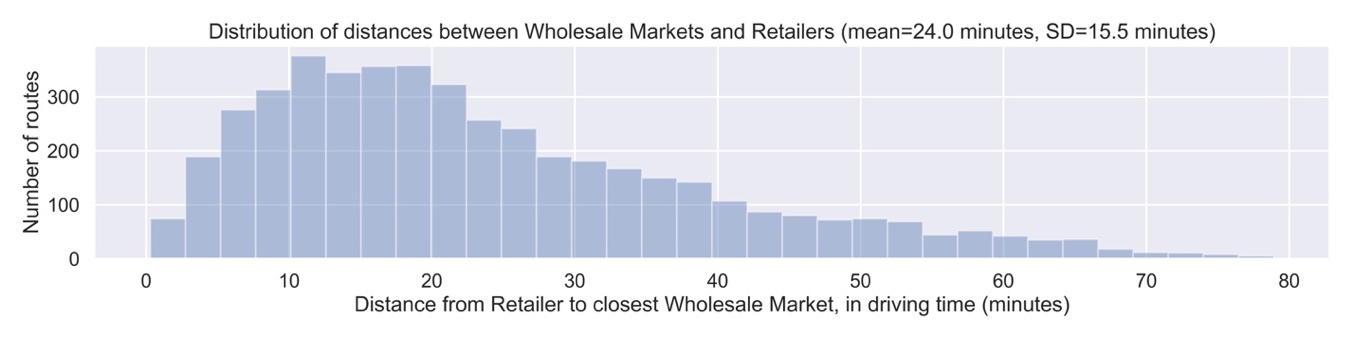  Figure S13 Distribution of travel times by route type. |

Vegetables from village markets proceeded to the closest wholesale market, which were an average of 27.4 minutes away (top histogram). Wholesale markets that traded with each other were an average of 4.8 hours away from each other (middle histogram). Retailers received vegetables from the closest wholesale market, which were an average of 24 minutes away (bottom histogram).

### 6.2.5 Type and quantity of vehicles for each route

For feasibility, each route type was assigned a single vehicle type: routes between village markets and wholesale markets were assigned single-axle trucks, routes between wholesale markets were assigned multi-axle trucks, and routes between wholesale markets and retailers were assigned motorbikes.

These vehicle types were based on market observations and input from key stakeholders, and they were triangulated against data from the Ministry of Road Transport and Highways. Of light motor vehicles used to transport goods (rather than passengers) in 2012, 20% were small 3-wheel trucks, 26% were small 4-wheel trucks, 47% were full-sized trucks and lorries, and 7% were multi-axle trucks.^33^ Although the Ministry of Road Transport and Highways does not designate motorbikes as “goods vehicles,” they are frequently used to transport smaller quantities of goods including food.

### 6.2.6 Vehicle Characteristics

Vehicle specifications, including capacity, fuel efficiencies, and capital and maintenance costs are shown in **Table S6.**

Table S6. Vehicle capacities

| Type of vehicle | Cargo capacity (liters) | Cargo capacity for lease dense product, cabbage (metric tons) | Cargo capacity for most dense product, potato (metric tons) |
| --- | --- | --- | --- |
| Motorbike | 500 | 0.2 | 0.3 |
| 3-wheel truck | 5,000 | 1.8 | 3.4 |
| Single-axle truck | 30,000 | 10.9 | 20.2 |
| Multi-axle truck | 50,000 | 18.1 | 33.4 |

Estimates of vehicle capacity were obtained by averaging cargo capacity volumes from vehicle specifications for various models in each category.^34^ Cargo capacity volumes for single-axle and multi-axle trucks were calculated by multiplying the length, width, and height of the cargo area, where the height of the cargo area was not limited to the height of the truck bed but matched the height of the passenger cabin, in order to represent moderate vehicle overloading. Cubic meters of cargo area were converted to liters. HERMES Agrifood assessed storage and transport capacity on the basis of volume. Because the capacity of vegetable trucks in India is frequently described on the basis of mass (e.g., 10 tons of potatoes), Table S6 also contains estimated probable bounds of mass based on the least dense product (cabbage, at 0.362 kg/L bulk density) and the most dense product (potato, at 0.675 kg/L bulk density). Because motorbikes do not have a cargo area for which to calculate volume, expert stakeholders provided estimates of the number of tons of each product that could be transported on a motorbike, and bulk densities of each crop were used to establish probable bounds of volume-based carrying capacity.

## 6.3 Vegetable Characteristics

Vegetables in the model were characterized by the amount supplied, the amount demanded, the space required, lifespans in ambient temperature storage and cold storage, and rates of loss.

### 6.3.1 Amount of vegetables entering the supply chain: geographic distribution between blocks

India’s 2011 Agriculture Census reported hectares under production for specific crops in each of Odisha’s 315 blocks,^35^ based on a sample of 20% of the villages in each block.^39^ Where there were missing block-level data in the agricultural census, zero production was assumed. Hectares under production were converted to tons of production using Odisha-specific average crop yields from the Government of India National Horticulture Board.^37^ Tons of production per block are shown for each crop in **Figure S14.**

| 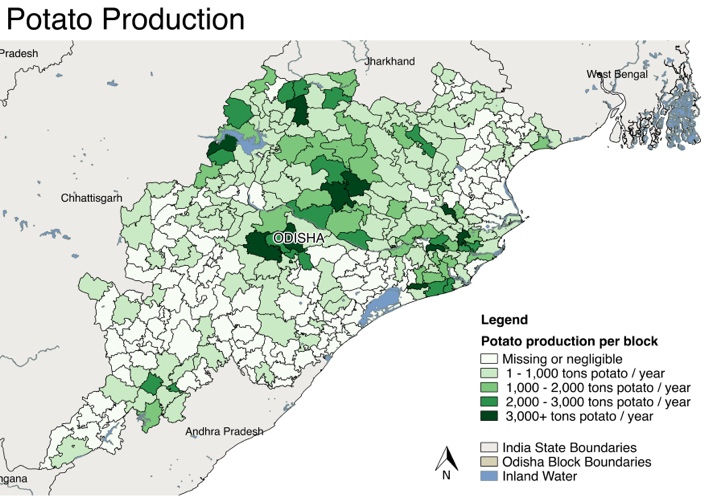 | 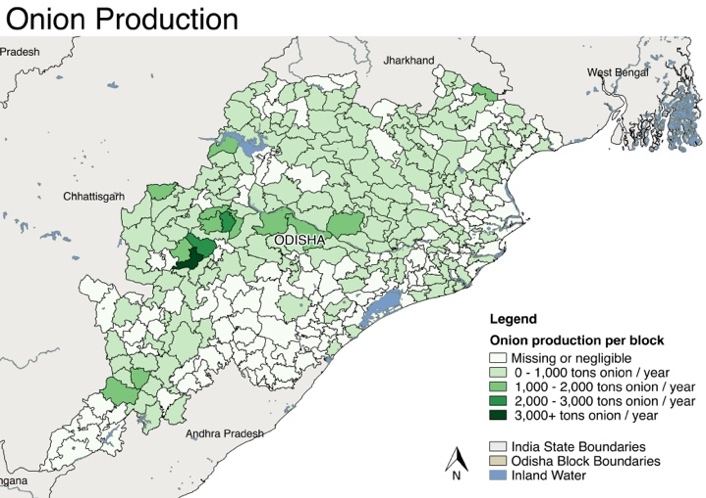 |
| --- | --- |
| 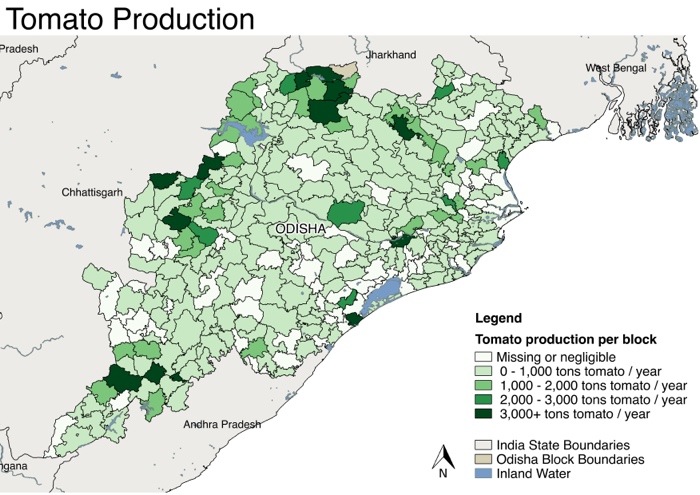 | 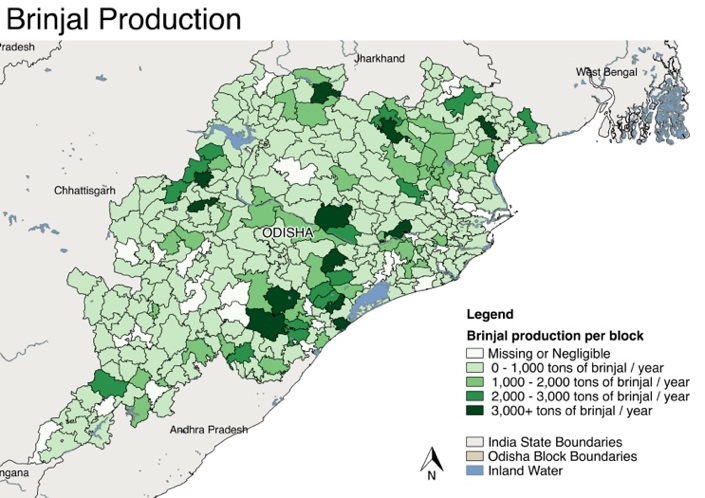 |
| 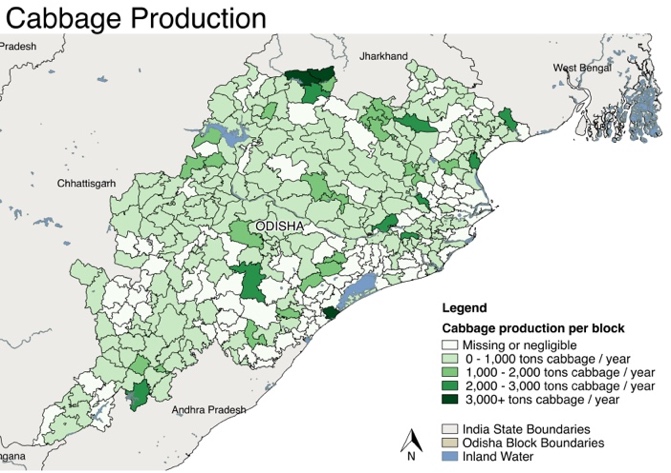  Figure S 14 Block-level production according to Agriculture Census data. | |

The block-level production values from the agriculture census were scaled up by multiplying factors to match estimates from the National Horticulture Board. The agricultural census data underestimate agricultural production for two major reasons: the estimates reflect only 20% of villages in each block,^39^ and horticultural crop production is difficult to track because vegetables are grown on smaller plots and are harvested frequently.^38^ More accurate estimates of production are available through the National Horticulture Board,^36^ but these estimates are available only at the district level (n=30), not at the block level (n=315). Based on a comparison of statewide average per capita production from both data sources (shown in **Figure S15**), multiplying factors ranging from 1.23 to 9.89 were applied to the block-level Agriculture Census values.

Figure S15 Comparison of per capita production and consumption of vegetables in Odisha.

Production estimates from the Agriculture Census combine data from the India Agriculture Census (hectares under production), Odisha-specific crop yields from the National Horticulture Board (tons per hectare), and 2011 India Demographic Census Data (population of Odisha). The Agriculture Census estimates are known to be low for horticultural crop production, whereas production estimates from the National Horticulture Board are higher, as shown here. Consumption estimates are from the National Sample Survey Office consumer expenditure data.

### 6.3.2 Amount of vegetables entering the supply chain: geographic distribution within blocks

Annual block-level production was divided equally among the village markets in each block, on the basis of mass (as opposed to volume or monetary value). **Figure S16** shows the distribution of number of village markets per block, which range from one to 78.


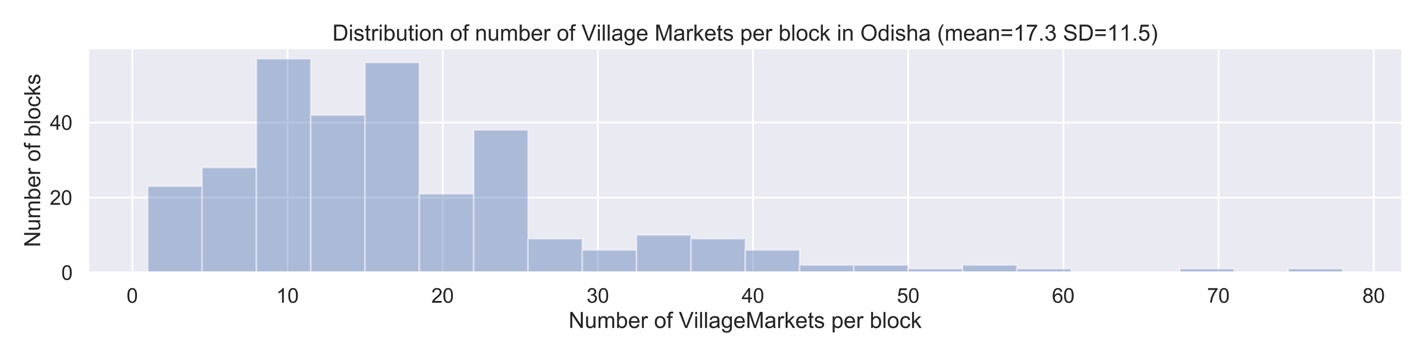


Figure S16 Distribution of number of village markets per block

### 6.3.3 Amount of vegetables entering the supply chain: temporal allocation between village markets

For village markets operating on a daily schedule (30.7% of the total), the amount of products entering the supply chain on each simulated operating day was equivalent to 1/336 of the annual amount, on the basis of mass. For village markets operating on a weekly schedule (69.3% of the total), the amount of product arriving on each simulated operating day was equivalent to 1/48 of the annual amount, on the basis of mass. In other words, weekly markets and daily markets in the same block received the same annual total amount of vegetables, but the size of the shipments on each operating day was larger in weekly markets by a factor of seven.

### 6.3.4 Amount of vegetables entering the supply chain: temporal allocation between seasons

The amount of products entering the supply chain through village markets varied by month according to the Odisha-specific seasonal patterns of harvest identified by the National Horticulture Board (NHB) in which there are peak-, lean-, and off-season months, as shown in **Figure S17.**^40^ During off-season months, there was zero production. During peak-season months, production was twice as high compared to lean-season months, on the basis of mass.

Figure S17 Seasonal calendar of vegetable harvest in Odisha from the National Horticulture Board.

To triangulate data from the seasonal calendar from the NHB, it was compared to wholesale market data from the AgMarknet system. As shown in **Figure S18**, for all five vegetables of interest, weekly market arrivals during months of peak production were on average 14% higher than weekly market arrivals during months of lean production and 15% higher than weekly market arrivals during months of no production. Although these differences between seasons were not statistically significantly different using a t-test, the trend towards higher wholesale transactions during months identified as “peak” by the NHB lend validity to the NHB seasonal calendar.

It’s worth noting that the monthly wholesale transaction data shown in Figure S18 do not distinguish between products grown in Odisha and products grown in other states. The fact that the data do not show dramatically lower transactions during months of lean or no production in Odisha suggest that wholesale markets are distributing foods grown outside of Odisha, which is reflected in our strategy of modeled “imports” and “exports” described in sections 6.3.5 and 6.3.6.

Figure S18 weekly wholesale market arrivals in Odisha, season seasons of peak and lean harvest.

The wholesale market arrivals data are from the AgMarknet database, and each bar shows average arrivals (in tons) for a week in 2017. The averages are from 106 reporting markets within the AgMarknet system. The classification of weeks of the year as peak-, lean-, or off-season is from the National Horticulture Board.

### 6.3.5 Imports: Meeting off-season demand through products from other states

For each crop, during any month in which the total amount produced within Odisha was not equal to or greater than the total amount demanded (based on per capita consumer expenditures^59^ multiplied by the total state population size^20^), products entered the supply chain through Tier 1 wholesale markets in amounts necessary to meet typical consumer demand, representing state-level “imports” of vegetables produced outside of the state of Odisha. During off-season months for any given crop, the entirety of demand was met from vegetables produced outside the state of Odisha.

### 6.3.6 Exports: Trading surplus products to other states

For each crop, during months in which typical production exceeded typical demand, products exited the supply chain through the largest Tier 1 wholesale market, representing state-level “exports” to other states.

### 6.3.7 Physical volume of vegetables

Crop production is typically reported on the basis of mass (kg or tons); because HERMES Agrifood packs storage devices by volume, crop-specific bulk densities were used to convert mass to volume.^64^ When possible, bulk densities for vegetables specific to India were used.^41^ The bulk densities are shown in **Table S7.**

Vegetable volume and mass can decrease over time due to shrinkage from moisture loss.^65^ In this model, shrinkage due to moisture loss was assumed to be unappreciable; this is especially true for the more perishable vegetables that generally pass through the supply chain in less than one week (tomato, brinjal, and cabbage). For the less perishable vegetables (potato, onion) it was assumed that any substantial moisture loss would contribute to rates of loss and waste which were accounted for through other model mechanisms (i.e., expiration or breakage).

### 6.3.8 Required cold storage conditions for vegetables

The model included two storage conditions: ambient temperature storage and cold storage. In this model, products did not *require* cold storage, but any time spent in cold storage slowed the rate of product degradation (see “factors that influence vegetable loss”).

### 6.3.9 Average lifespan of vegetables at various storage conditions

Each product in the model had an average lifespan at each storage condition (ambient storage or cold storage), as shown in **Table S7.** These lifespans were informed by empirical studies on the shelf life of potato,^42,44-46,52^ onion,^42,45,52,53^ tomato,^42-44,47,49,51,52^ brinjal,^42,44,48,52^ and cabbage^42,45,50,52^ at various storage conditions. When possible, studies conducted in India were used.

Table S7. Vegetable characteristics: demand, bulk density, and lifespan

| Product | Bulk density, in kg/ml | Average lifespan in ambient temperature storage, in days | Average lifespan in cold storage, in days | Annual per capita expenditures in Odisha, in kg / capita / year |
| --- | --- | --- | --- | --- |
| Potato | 0.675 | 21 | 180 | 28.9 |
| Onion | 0.643 | 15 | 150 | 9.4 |
| Tomato | 0.481 | 6 | 10 | 8.9 |
| Brinjal | 0.501 | 4 | 9 | 9.6 |
| Cabbage | 0.362 | 7 | 30 | 4.2 |

### 6.3.10 Prioritization of vegetables in storage devices

When attempting to pack a storage device of limited capacity, priority was given to vegetables with a shorter maximum lifespan, in order to prioritize more perishable vegetables. This applied to storage devices at both ambient temperature and cold storage.

For example, if 100 liters of potato and 100 liters of tomato were awaiting packing onto a vehicle with a maximum capacity of 150 liters of ambient temperature, because tomato has a shorter maximum lifespan the vehicle would be packed with 100 liters of tomato and 50 liters of potato, and the remaining 50 liters of potato would await the next shipment. The next shipment might occur the following week, the following day, or later in the same day, depending on the operating schedules described in section 6.1.5 and the route types shown in Table S5. For example, for village markets operating on a daily schedule, trucks transporting vegetables to wholesale markets would take multiple trips per day, so vegetables not included in the first shipment would have an opportunity to be shipped later in the same day.

### 6.3.11 Factors that influence vegetable loss

The model represented total vegetable loss as the sum of two types of loss: expiration and breakage.

**Expiration** refers to product losses that occurred when a product reached the end of its lifespan before being purchased by a consumer or experiencing breakage. Average lifespans are shown in **Table S7.** Because crops had longer lifespans in cold storage, time spent in cold storage slowed products’ aging rates. As time progressed in any given simulation, each crop proceeded towards its maximum lifespan at a linear rate depending on its storage condition, and expiration would occur once the product’s age exceeded its maximum lifespan. Aging rates were updated each time a product was packed or unpacked. Expiration therefore represented both suboptimal temperature storage conditions and an excessive amount of time in the supply chain.

**Breakage** refers to unavoidable product loss that occurred during storage and transport, such as loss from unavoidable factors (e.g., accidental spills) and typical conditions of transportation, packaging, and handling. The breakage rates were Poisson-distributed, with the mean breakage rates set at 2% at each supply chain level and 2% during each leg of transport. This adds up to approximately 12% unavoidable breakage. These breakage rates were informed by literature sources that measured postharvest loss by supply chain level, taking into account typical styles of transportation, packaging, and handling in India.^4,6,13,54-58^ For the five crops of interest, these sources found that average storage losses at the wholesale level ranged from 2.7% to 8.8%, average storage losses at the retail level ranged from 3.0% to 6.6%, and losses during transport by traders ranged from negligible to 6.4%. Many of these studies’ methods are known to lead to under-estimation, as they are based on survey and observational data.

## 6.4 Consumer Demand for Vegetables

### 6.4.1 Types of people

HERMES Agrifood represented one population type. Distinctions were not made between the demand of people of different age categories or sexes because quantities of typical demand were based on consumer expenditure data that averaged all population groups.^59^ It was assumed that adults in the model demanded the same amount of product each day, and that demand did not vary between rural and urban areas.

### 6.4.2 Quantity of vegetables demanded per person

The quantity of vegetables demanded per person was based on Odisha-specific per capita expenditures (in kilograms per person) for specific food commodities from the Government of India National Sample Survey Office,^59^ as shown in **Table S7.** The annual per capita expenditures shown in the table are averages between rural and urban expenditures.

It should be noted that rural areas in Odisha typically see higher per capita expenditures for potato, and urban areas in Odisha typically see higher per capita expenditures for onion, tomato, brinjal, and cabbage. Consumer expenditure data are an imperfect proxy for consumer demand because they reflect a combination of both quantity and price, but expenditure data were used because they were available on a state- and commodity-specific basis at the household level. Data on direct amounts of consumption (through recall-based dietary assessment methods) are available through sources such as the Ministry of Health and Family Welfare’s National Family Health Survey, but are generally not available disaggregated by state or commodity.

### 6.4.3 Quantity of people expected at each location

Simulated consumers could purchase vegetables from village markets, wholesale markets, and retailers. The timing of the consumer arrivals at these locations drew from a Poisson distribution. The size of the simulated consumer population for each location was determined by calculating the number of people within the catchment area for each village market, wholesale market, or retailer, based on population count data from the Gridded Population of the World dataset^23^ and geolocations for each supply chain level. **Figure S19** shows the distribution of populations served by locations at each supply chain level.

Village Markets: At village markets, 5% of the catchment area arrived each day to represent purchases from nearby residents. The size of this “local purchase” population (5% of the catchment area) was an average of 390 people (SD=377 people) at village markets throughout Odisha. Products not purchased by these local residents proceeded through the supply chain to wholesale markets.

Wholesale Markets: At wholesale markets within the “focus region” shown in Figure S1, 5% of the catchment area arrived each day to represent the “local purchase” population, and the remaining products proceeded through the supply chain to be traded with other wholesale markets or to supply individual retailers in the area. For wholesale markets inside the focus region, the average local purchase population was 5,464 people (SD=5,202 people).

Outside of the “focus region” shown in Figure S1, 5% of the catchment area represented the “local purchase” population and the demand of the remaining 95% of the catchment area was represented by a single “retail surrogate.” Rather than representing all retailers outside the focus region as individual modeled entities, we represented the demand for the population served by these retailers with a single retail surrogate location supplied by each wholesale market. This allowed us to simulate the same volume of product passing through wholesalers to the same amount of population demand for the accompanying retailers, without the substantially longer simulation run times that would have resulted from representing all retailers individually.^[[4]](#footnote-4)^ Remaining products not consumed by the catchment area proceeded through the supply chain to be traded with other wholesale markets. For wholesale markets outside the focus region, the average retail surrogate population was 103,000 people (SD=99,000 people), which represents the aggregate demand for all retailers that would theoretically fetch their products from a given wholesale market.

Retailers: At the retail level, the remaining portion of each catchment area not served directly by Village Markets or Wholesale Markets arrived at each retail location on each simulation day. While in reality not all people visit a vegetable retailer each day, this method approximates the average magnitude of daily demand from people in the catchment area. Survey research with consumers in urban areas of Odisha showed that 17% of consumers made vegetable purchases on a daily basis, 32% made vegetable purchases 3-6 days per week, and 46% made vegetable purchases 2 days per week.^66^

| 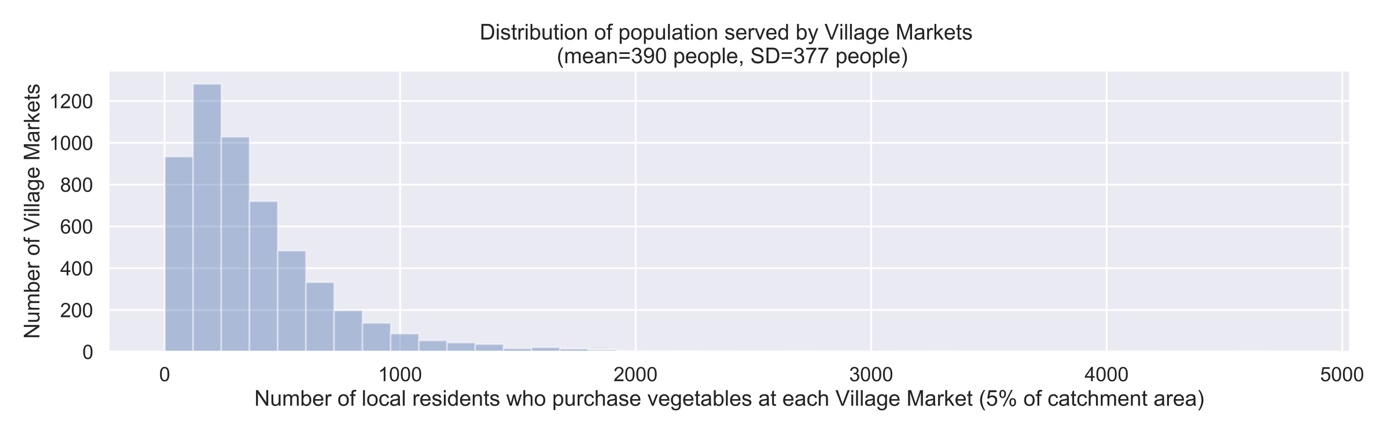 |
| --- |
| 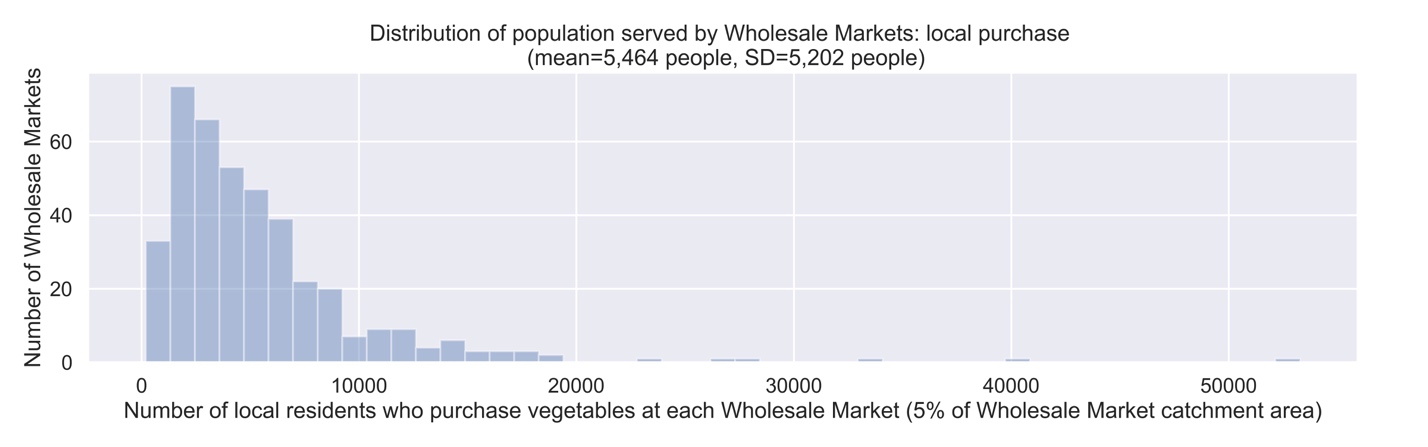 |
| 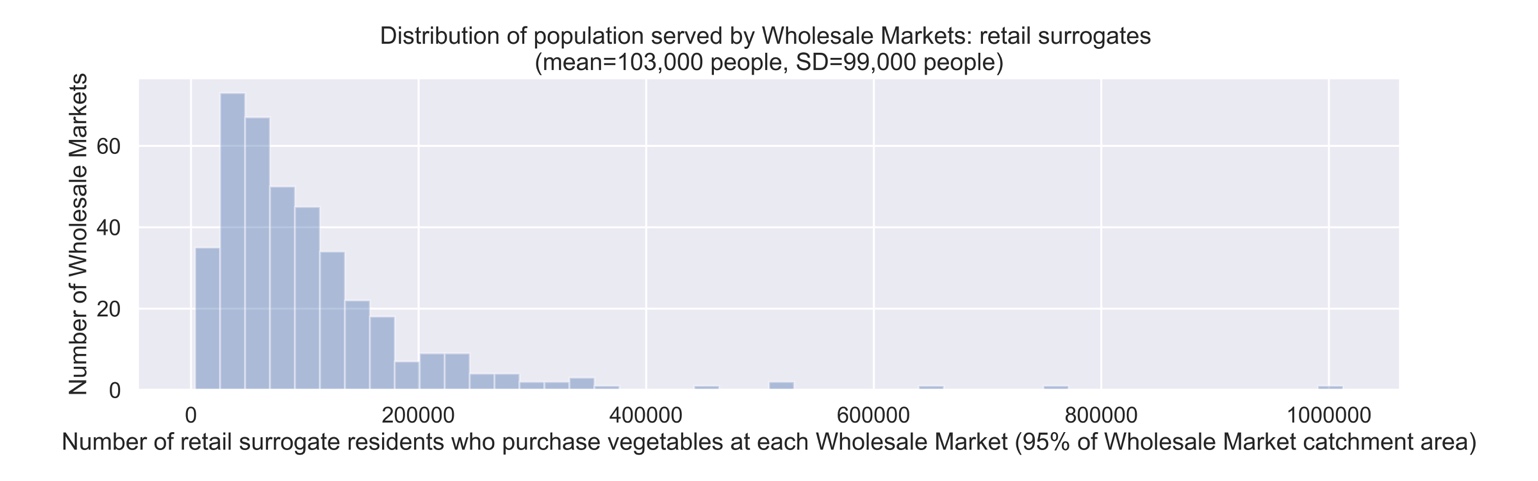  Figure S19 Distribution of population served by types of supply chain locations. |

*Catchment areas (number of people served by any given supply chain location) were calculated using population counts per square kilometer from the Gridded Population of the World dataset and geolocations of supply chain locations. For retailers, 95% of the catchment area arrived each day to purchase vegetables. For village markets or wholesale markets, 5% of the catchment area arrived to purchase vegetables, and the remaining vegetables proceeded through the supply chain.*

# Supplemental References

1. Government of India, Ministry of Home Affairs, India Census Bureau. India Census 2011. <http://www.censusindia.gov.in/>. Published 2011. Accessed August 1, 2017.

2. Chanda S. *The Border Society: A Study of Network and Linkage.* Kolkata, India: Anthropological Survey of India; 2003.

3. Sivaramane N, Reddy G. *Supply Chain Management in Agriculture.* Telangana, India: National Academy of Agricultural Research Management (NAARM); 2014.

4. Sharma G, Singh S. Economic analysis of post-harvest losses in marketing of vegetables in Uttarakhand. *Agricultural Economics Research Review.* 2011;24(2):309-315.

5. Suvasmita A. *Marketing Analysis of Vegetables in Khorda District Under OCTMP*. Bhubaneswar, Odisha: Department of Agribusiness Management, Orissa University of Agriculture and Technology; 2016.

6. Marandi MK. *Economics of Vegetable Production and Marketing in Lahunipara Block of Sundargarh District*. Bhubaneswar, Odissha: Agricultural Economics, Orissa University of Agriculture and Technology; 2013.

7. Dastagiri M. *Estimation of Marketing Efficiency of Horticultural Commodities under Different Supply Chains in India.* New Delhi: National Centre for Agricultural Economics and Policy Research;2010.

8. Hegde R, Madhuri N. A study on marketing infrastructure for fruits and vegetables in India. *National Institute of Rural Development.* 2013;91.

9. Kumar NRR, Rajesh K. Analysis of Supply Chain Mangement in Potato Export from India. *Indian Journal of Agricultural Marketing.* 2009;23(1):10-19.

10. Negi S, Anand N. Issues and Challenges in the Supply Chain of Fruits & Vegetables Sector in India: A Review. *International Journal of Managing Value and Supply Chains.* 2015;6(2):47-62.

11. Rais M, Sheoran A. Scope of Supply Chain Management in Fruits and Vegetables in India. *Journal of Food Processing & Technology.* 2015;6(3):427.

12. Singh S. Farm to Fork Supply Chain of Potato in the State of Bihar in India. *Centre for Civil Society Working Paper Series.* 2011(252).

13. Saha SS, Chimalwar SP, Sherkar SB. Post Harvest Profile of Potato. *Government of India, Directorate of Marketing and Inspection.* 2007.

14. Díaz-Emparanza I. Is a small Monte Carlo analysis a good analysis? *Statistical Papers.* 2002;43(4):567-577.

15. Haidari LA, Brown ST, Wedlock P, Lee BY. Map of different vaccine supply chain efficiency measures. *Vaccine.* 2017;35(1):199-200.

16. Global Administrative Areas (GADM). GADM Database of Global Administrative Areas: India. <http://www.gadm.org/country>. Published 2015. Accessed August 11, 2017.

17. India Map Store. <https://store.mapsofindia.com/>. Published 2017. Accessed August 1, 2017.

18. Google. Google Maps Application Programming Interface (API). Published n.d.

19. Google. Google Directions Application Programming Interface (API). n.d.

20. Government of India, Ministry of Home Affairs, India Census Bureau. *India Census 2011: District Census Handbook for Odisha.* 2011.

21. Government of India, Ministry of Agriculture & Farmers Welfare, Directorate of Marketing and Inspection (DMI). Directory of Wholesale Agricultural Produce Assembling Markets in India. <http://agmarknet.gov.in/Others/dwapdir.pdf>. Published 2004. Accessed August 9, 2017.

22. Government of India, Ministry of Agriculture & Farmers Welfare, Directorate of Marketing and Inspection (DMI). AgMarknet Prices & Arrivals Online Database. <http://agmarknet.gov.in/PriceTrends/>. Published 2017. Updated July 26, 2017.

23. Center for International Earth Science Information Network - CIESIN - Columbia University. Gridded Population of the World, Version 4 (GPWv4): Population Count. In. Palisades, NY: NASA Socioeconomic Data and Applications Center (SEDAC); 2016.

24. Srivastava R. Changing retail scene in India. *International Journal of Retail & Distribution Management.* 2008;36(9):714-721.

25. Government of Odisha, Department of Agriculture and Farmers Empowerment, Directorate of Horticulture, Orissa Horticulture Development Society (OHDS). Project Report on 10MT Cool Chamber. <http://odihort.nic.in/nhm-schemes>. Published 2012. Accessed August 11, 2017.

26. National Centre for Cold Chain Development. All India Cold-chain Infrastructure Capacity Assessment of Status & Gap. <http://www.nccd.gov.in/PDF/CCSG_Final%20Report_Web.pdf>. Published 2015.

27. National Centre for Cold-Chain Development. *Cold Chain Development for Fruits & Vegetables in India: Kinnow Cold Chain Study.* 2016.

28. Winrock International. Empowering Agriculture: Energy Options for Horticulture. In. United States Agency for International Development (USAID) 2009.

29. Kitinoja L, Thompson JF. Pre-cooling systems for small-scale producers. *Stewart Postharvest Review.* 2010;2(2).

30. Wilbur Smith Associates Private Limited. *Study on Traffic and Transportation Policies and Strategies in Urban Areas in India (prepared for the Government of India, Ministry of Urban Development).* 2008.

31. Alam M, Ahmed F. Urban transport systems and congestion: a case study of indian cities. *Transport and Communications Bulletin for Asia and the Pacific.* 2013;82:33-43.

32. Pal S, Roy SK. Impact of Roadside Friction on Travel Speed and LOS of Rural Highways in India. *Transportation in Developing Economies.* 2016;2(2):9.

33. Government of India, Ministry of Road Transport & Highways. *Road Transport Year Book (2013-14 and 2014-15).* New Delhi, India. 2015.

34. TrucksDekho. Vehicle Specifications. <https://trucks.cardekho.com/>. Published 2016.

35. Government of India, Agriculture Census Division. Agriculture Census, Table 6B: Estimated Irrigated And Unirrigated Area By Size Classes Under Crop. <http://agcensus.dacnet.nic.in/TalukCharacteristics.aspx>. Published 2011.

36. Government of India, Ministry of Agriculture & Farmers’ Welfare, National Horticulture Board. Horticulture Crops Estimate for the Year 2011-12. Published 2012. Accessed December 6, 2018.

37. Government of India, Ministry of Agriculture & Farmers’ Welfare, National Horticulture Board. Yield Comparison of Horticulture Crops State Wise. Published 2015. Accessed April 30, 2018.

38. Ahmad T. Module 1.7: Horticulture Surveys in Module 1, "Sample Techniques and Agricultural Survey". In: Prachi Misra Sahoo, Tauqueer Ahmad, Anil Rai, K.N. Singh, Sud UC, eds. *Applications of Remote Sensing and GIS in Agricultural Surveys.* New Delhi, India: Indian Agricultural Statistics Research Institute (ICAR); 2003.

39. Sud U. Module 1.6: Agriculture Census in Module 1, "Sample Techniques and Agricultural Survey". In: Prachi Misra Sahoo, Tauqueer Ahmad, Anil Rai, K.N. Singh, Sud UC, eds. *Applications of Remote Sensing and GIS in Agricultural Surveys.* New Delhi, India: Indian Agricultural Statistics Research Institute (ICAR); 2003.

40. Government of India, Ministry of Agriculture & Farmers’ Welfare, National Horticulture Board. *Indian Horticulture Database 2014.* New Delhi. 2014.

41. Sharan G, Rawale K. *Physical Characteristics of Some Vegetables Grown in Ahmedabad Region.* Indian Institute of Management Ahmedabad, Research and Publication Department;2003.

42. Government of India, Ministry of Agriculture & Farmers Welfare, Horticulture Statistics Division. *Horticultural Statistics at a Glance 2017.* 2017.

43. Indian Council of Agricultural Research. *Impact of Vegetable Research in India.* New Delhi. 2004.

44. Rayaguru K, Khan MK, Sahoo N. Water use optimization in zero energy cool chambers for short term storage of fruits and vegetables in coastal area. *Journal of Food Science and Technology.* 2010;47(4):437-441.

45. Ali Z, Yadav A, Stobdan T, Singh SB. Traditional methods for storage of vegetables in cold arid region of Ladakh, India. *Indian Journal of Traditional Knowledge.* 2012;11(2):351-353.

46. Eltawil MA, Samuel DK, Singhal O. Potato storage technology and store design aspects. *Agricultural Engineering International: CIGR Journal.* 2006.

47. Dadhich SM, Dadhich H, Verma R. Comparative study on storage of fruits and vegetables in evaporative cool chamber and in ambient. *International Journal of Food Engineering.* 2008;4(1).

48. Ganesan M, Balasubramanian K, Bhavani R. Studies on the application of different levels of water on Zero energy cool chamber with reference to the shelf-life of brinjal. *J Indian Inst Sci.* 2004;84:107-111.

49. Nasrin T, Molla M, Hossaen MA, Alam M, Yasmin L. Effect of postharvest treatments on shelf life and quality of tomato. *Bangladesh Journal of Agricultural Research.* 2008;33(4):579-585.

50. Jiang T, Pearce D. Shelf-Life Extension of Leafy Vegetables: Evaluating the Impacts. In. Canberra and Sydney: Australian Center for International Agricultural Research (ACIAR); 2005.

51. Arah IK, Ahorbo GK, Anku EK, Kumah EK, Amaglo H. Postharvest handling practices and treatment methods for tomato handlers in developing countries: A mini review. *Advances in Agriculture.* 2016;2016.

52. Liberty J, Okonkwo W, Echiegu E. Evaporative cooling: A postharvest Technology for fruits and vegetables preservation. *International Journal of Scientific & Engineering Research.* 2013;4(8):2257-2266.

53. Ilić Z, Milenković L, Djurovka M, Trajković R. The effect of long-term storage on quality attributes and storage potential of different onion cultivars. Paper presented at: IV Balkan Symposium on Vegetables and Potatoes, 2008.

54. Jha S, Vishwakarma R, Ahmad T, Rai A, Dixit A. Report on Assessment of Quantitative Harvest and Post-Harvest Losses (Repeat Study). In: Indian Council for Agriculture Research (ICAR), Central Institute of Post-Harvest Engineering and Technology (CIPHET), eds. Ludhiana. 2015.

55. Narayana C, pandey B, Malhotra S, Pandey V. *Post Harvest Losses in Selected Fruits and Vegetables in India (A Compilation).* Bengaluru, India: Indian Institute of Horticultural Research, Technical Bulletin 41;2014.

56. Dahiya PS, Khatana V, Ilangantileke S, Dabas J. Potato storage patterns and practices in Meerut district, Western Uttar Pradesh, India. *Working Paper (CIP).* 1996.

57. Kitinoja L, Kader AA. *Measuring postharvest losses of fresh fruits and vegetables in developing countries.* The Postharvest Education Foundation; 2015.

58. Kumar N, Pandey N, Dahiya P, Rana R, Pandit A. Post Harvest Losses of Potato in West Bengal: An Economic Analysis. *Potato Journal.* 2004;31(3-4).

59. Government of India, Ministry of Statistics and Programme Implementation, National Sample Survey Office. *Household Consumption Expenditure, National Sample Survey Office (NSSO) 68th Round, 2011-2012.* 2012.

60. QGIS Development Team. QGIS Geographic Information System. Open Source Geospatial Foundation Project. <http://qgis.osgeo.org>. Published n.d. Accessed August 1, 2017.

61. Rajkumar P, Jacob F. Business models of vegetable retailers in India. *Great Lakes Herald.* 2010;4(1):31-43.

62. Finzer LE, Ajay VS, Ali MK, et al. Fruit and vegetable purchasing patterns and preferences in South Delhi. *Ecology of food and nutrition.* 2013;52(1):1-20.

63. Government of India, Ministry of Agriculture & Farmers Welfare, Directorate of Marketing and Inspection (DMI). Estimation of Marketable Surplus & Postharvest Losses of Foodgrains in India: Methodology and Profile of Sample Villages. In:2002.

64. Beukema KJ. *Heat and mass transfer during cooling and storage of agricultural products as influenced by natural convection*, Wageningen University; 1980.

65. Lozano J, Rotstein E, Urbicain M. Shrinkage, porosity and bulk density of foodstuffs at changing moisture contents. *Journal of food Science.* 1983;48(5):1497-1502.

66. Kapoor S, Kumar N. Fruit and Vegetable Consumers’ Behavior: Implications for Organized Retailers in Emerging Markets. *Journal of International Food & Agribusiness Marketing.* 2015;27(3):203-227.

1. This number was calculated by dividing the total annual tons of arrivals for potato, onion, tomato, brinjal, and cabbage by the total annual tons of all commodities at the 106 wholesale markets within the AgMarknet reporting system in Odisha in 2017. The AgMarknet system is maintained by the Government of India Directorate of Marketing & Inspection. [↑](#footnote-ref-1)
2. Representing individual retail locations for all of Odisha (including outside of the focus region) would require the addition of over 110,00 retailer locations to the model, based on an estimated density of 1 vegetable retailer for every 400 residents and a state population of 44 million, which would contribute to longer model run times. It is for this reason that retail surrogates were used outside of the focus region. See section 6.5.3 for more detail. [↑](#footnote-ref-2)
3. In some cases, a single geographic location could be represented by more than one computational entity, in order to represent distinct functions. For example, each wholesale market used one entity to distribute products through the supply chain, and a separate entity to sell products to consumers. [↑](#footnote-ref-3)
4. Representing all retailers throughout the whole state of Odisha individually would have required the addition of over 110,000 model entities, based on an estimated density of 1 vegetable retailer for every 400 residents (see section 6.1.4 for more detail) and a state population of 44 million. The simulation runs conducted here included 10,543 distinct locations represented by 21,855 computational entities (see section 4.3 for more detail); adding 110,000 entities would have substantially increased simulation run time. [↑](#footnote-ref-4)
